# Rolling down that mountain: microgeographical adaptive divergence during a fast population expansion along a steep environmental gradient in European beech

**DOI:** 10.1038/s41437-024-00696-z

**Published:** 2024-06-18

**Authors:** Andrea Modica, Hadrien Lalagüe, Sylvie Muratorio, Ivan Scotti

**Affiliations:** 1 INRAE, URFM, 228, Route de l’Aérodrome, 84914, Avignon, France; 2 INRAE, EcoFoG, Campus agronomique, 97310 Kourou, French Guiana; 3 INRAE, EcoBioP, 173, Route de Saint-Jean-de-Luz RD 918, 64310 Saint-Pée-sur-Nivelle, France

**Keywords:** Genetic variation, Ecological genetics

## Abstract

Forest tree populations harbour high genetic diversity thanks to large effective population sizes and strong gene flow, allowing them to diversify through adaptation to local environmental pressures within dispersal distance. Many tree populations also experienced historical demographic fluctuations, including spatial population contraction or expansions at various temporal scales, which may constrain their ability to adapt to environmental variations. Our aim is to investigate how recent contraction and expansion events interfere with local adaptation, by studying patterns of adaptive divergence between closely related stands undergoing environmentally contrasted conditions, and having or not recently expanded. To investigate genome-wide signatures of local adaptation while accounting for demography, we analysed divergence in a European beech population by testing pairwise differentiation among four tree stands at ~35k Single Nucleotide Polymorphisms from ~9k genomic regions. We applied three divergence outlier search methods resting on different assumptions and targeting either single SNPs or contiguous genomic regions, while accounting for the effect of population size variations on genetic divergence. We found 27 signals of selective signatures in 19 target regions. Putatively adaptive divergence involved all stand pairs. We retrieved signals both when comparing old-growth stands and recently colonised areas and when comparing stands within the old-growth area. Therefore, adaptive divergence processes have taken place both over short time spans, under strong environmental contrasts, and over short ecological gradients, in populations that have been stable in the long term. This suggests that standing genetic variation supports local, microgeographic divergence processes, which can maintain genetic diversity at the landscape level.

## Introduction

The patterns of genetic diversity currently displayed by wild populations are the result of multiple demographic, ecological and evolutionary processes triggered by historical events, such as long-term climatic cycles, disturbances, and land-use change by humans. Understanding and/or predicting patterns of genome diversity requires unveiling the complex interactions between the demographic fluctuations caused by historical contingencies, on the one hand, and the intrinsic capacity of populations to evolve in response to these contingencies, on the other hand (e.g., selection is less effective, and migration more effective, in smaller populations, Hedrick 2000). Conversely, evolutionary forces can shape population demography (e.g., inbreeding can depress fecundity, Charlesworth and Charlesworth 1987; hard selection (i.e., the situation in which an individual’s fitness depends only on its own phenotype) may reduce population size; Bell et al. 2021). Such feedback loops are often hard to tease apart, and this can be particularly challenging for long-lived species playing key ecological roles, such as widespread forest trees. Indeed, their often large populations can persist over tens of millennia, involving complex demographic histories, marked by range shifts, long stretches of time in isolation from other populations, multiple disturbance regimes and the ever-present danger posed by anthropogenic deforestation (Petit and Hampe 2006). Yet, deconvoluting the intricate roles of demographic and selective histories on tree population diversity and structure is all the more urgent in view of the ongoing biodiversity crisis, which calls for the implementation of adaptive management or/and conservation strategies for forest ecosystems, benefitting from and/or caring for intraspecific diversity and evolutionary processes (Godineau et al. 2023).

A wide range of methods have been developed in the last decades to infer population demographic (reviewed in Beichman et al. 2018) or selective histories (reviewed in Nielsen 2005; Bank et al. 2014) from patterns of genomic diversity. Across non-model taxa, and specifically in trees, the predominant approaches used for uncovering sets of loci underlying local adaptation have neglected explicit demography (Lind et al. 2018). These approaches generally rely on population divergence patterns and can be roughly divided into two categories: (1) those relying on the partition of between-population genetic variance (resting on the approach originally formulated in Lewontin and Krakauer 1973) to identify loci showing unusually high or low between-population differentiation (typically measured by *F*_ST_) and (2) those relying on the regression of population allele frequencies onto one or more environmental variables to identify loci showing strong correlations (i.e., gene-environment association, GEA). In the first category, some explicit variants of the island model (including hierarchical versions) were historically used to obtain neutral *F*_ST_ expectations against which to identify divergence outliers (e.g., Foll et al. 2008). More recently, for both categories, more flexible, less model-based parameterisation approaches have been used, where the null model generally assumes that the allele frequencies in each population may deviate away from ancestral (or global) allele frequencies due to genetic drift and gene flow, and where this covariance structure is accounted for to estimate divergent selection (e.g., Coop et al. 2010; Gautier 2015). Although these different approaches to detect divergent selection intentionally avoid using any explicit model of the historical relationships between populations, they were shown to be remarkably robust to demographic history (De Villemereuil et al. 2014; Lotterhos and Whitlock 2014). At the same time, many genomic studies confirmed that the current level and distribution of genetic diversity of tree populations show pervasive signatures of demographic fluctuations, be they ancient (such as the well-studied post-glacial expansion history, Petit et al. 2003) or recent (Zhao et al. 2020). A major risk when studying adaptation in recently expanded populations is that the marks left by demographic events on genomic patterns of diversity can mimic adaptive patterns. The classic case is allele surfing, when a neutral allele increases in frequency across the expansion range due to genetic drift and founder effect (Excoffier and Ray 2008; Excoffier et al. 2009). A typical example of the combined effect of range / demographic expansion and concurrent adaptation on population genetic structure is provided by Zhao et al. (2020), who manually removed geographically structured loci from the list of loci showing association with environmental variables. In addition, new adaptations arising during expansion can contribute to the success of the expansion itself, as shown in the case of the invasive plant, *Lythrium solicaria* L., by Colautti and Barrett (2013). Importantly, it is likely that the distribution of genetic diversity in recently expanded populations is far from any migration-selection or migration-selection-drift equilibrium. The incorporation of the demographic background in any population model describing such non-equilibrium situations is thus essential: any inference of the selective history should explicitly test selection against the backdrop of ongoing or recent demographic changes. Any conclusion drawn from the analysis of such “space with time” models is likely to provide more realistic predictions on how climate change will affect patterns of local adaptation than do the standard “space for time” approaches resting on stable, supposedly fully locally adapted populations (Hansen et al. 2012).

In this study, we advocate for a generalised approach to the incorporation of detailed demographic models, while taking into account the uncertainties of the estimation of demographic model parameters, into the search for divergence outliers. The approach we propose involves the generation of simulated neutral distributions of the chosen divergence statistic, based on the demographic model that has been previously determined, followed by the comparison of the empirical values of the statistic with the simulated distribution. Myers et al. (2008) suggest that a given genomic data set is compatible with large numbers of alternative demographic scenarios, and Lapierre et al. (2017) suggest that small errors in genomic data can lead to major biases in demographic inference. By simulating data from a range of estimates of demographic parameters, our method accounts for the uncertainties associated with demographic inferences from allele frequency data; because such simulations can be performed for any demographic model, data set, and divergence statistic, the method is general.

To investigate the joint demographic and selective histories in recently expanded tree populations, we focused on relatively common situations encountered in European areas that were densely populated by humans in the (pre)historic past. A general trend exists in Europe, with deforestation steadily reducing forest cover from the Holocene until the industrial revolution, followed by a partial reversal of this trend starting mid-nineteenth century (Kaplan et al. 2009; Zanon et al. 2018). The slopes of Mont Ventoux, in southern France, are a typical example, where anthropogenic pressure has been increasing since the Neolithic, throughout historical times, until it tapered off between the end of the nineteenth century and mid-twentieth century. From 1860, a massive rural flight, locally accompanied by measures aiming at reforestation and at supporting natural forest regeneration, favoured the expansion of forest remnants into newly thriving forests (Ningre 2007; Martin-Gousset et al. 2019; Gamba et al. 2023). The remnants of beech stands having persisted in historical times were restricted to high-elevation forest patches, and they gradually colonised land at lower elevations, thus meeting conditions that are both warmer and wetter. Our hypothesis is that such population expansions involved both demographic changes and adaptive processes, even on these restricted spatial scales, causing a reshuffling or filtering of genetic diversity within the population. In agreement with these differential selection pressures, signatures of local adaptation were identified for growth and phenological characters (Gauzere et al. 2020) based on a quantitative genetic approach. Moreover, a candidate-gene study suggested adaptive divergence at the molecular level (Csilléry et al. 2014). Such characteristics make it likely that the recolonisation process has left genomic signatures of microgeographic adaptation, and it should therefore be possible to identify genetic loci showing divergence both between sub-sections of the historical relict areas, historically exposed to different environmental conditions (e.g., north- vs south-facing slopes), and between historical relict and recently colonised areas.

The present study takes advantage of this well-studied population to investigate genome-wide signature of local adaptation while accounting for demography. We build on the extant knowledge about the Mont Ventoux beech population history, which was previously investigated based on palaeocological and historical records as well as neutral genetic diversity (Lander et al. 2011, 2021; Gamba et al. 2023). This exceptional knowledge of the recent demography of the study population made it possible to finely calibrate a selection inference method accounting for population demography. Another major asset on which this study relies is the recent establishment of a high-quality reference genome sequence for *F. sylvatica* (Mishra et al. 2022). Studies relying on annotated forest tree genomes are so far restricted to few species (*Pinus taeda* L., Lu et al. 2019; De La Torre et al. 2019; *Quercus robur* L., Leroy et al. 2020; *Populus* spp., Liu et al. 2022).

**Fig. 1:**
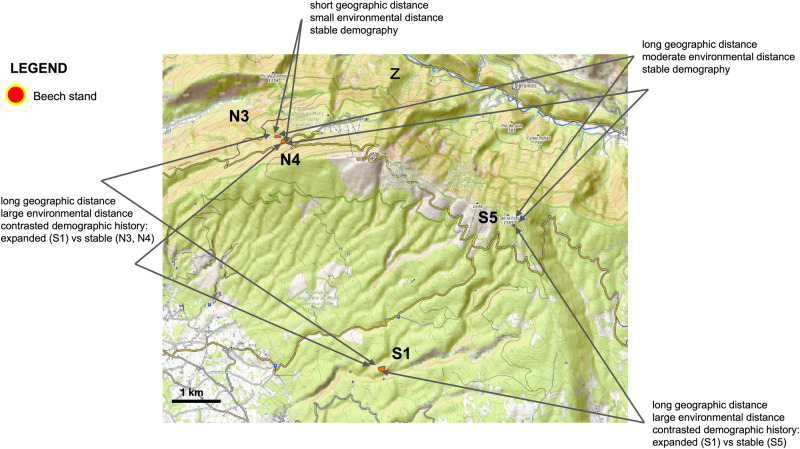
Qualitative description of combinations of levels of temporal, spatial, and environmental divergence for population pairs. Notice that “long” and “short” should be interpreted in a landscape-level context, not to be confused with processes occurring at larger divergence scales (regional, rangewide, phylogenetic). See Materials and Methods and Table 1 for the detailed description of the sampled stands.

To carry out our study, we focused on single-copy gene regions of the *F. sylvatica* reference genome sequence (Mishra et al. 2022), and on four stands belonging to the Mont Ventoux beech population (three in historical relict areas and one in a recently colonised area). These stands show varying degrees of divergence in environmental conditions (Table 1; see Wells et al. (2004), Davi and Cailleret (2017) for original data and definition of indices), and lie at varying spatial distances. The combination of these three variables leads to different expectations on adaptive divergence patterns, depending on the strength of selective pressures in time and space (Fig. 1). Our results show (adaptive) molecular divergence at all hierarchical levels of population subdivision.

**Table 1:** Characteristics of the stands; all measurements refer to years 1959–1990 (Muratorio et al. in prep.).

Stand	origin	Slope	Elevation (m a.s.l.)	Tmax (°C)	Tmin (°C)	sum of GDD (d)	NFD (d)	VPD May to September (mbar)	stress May to September (mm)	scPDSI May to September (adimensional)
N3	Refugial	N	1226	20.4	−2.0	2496	27	414.1343	102.5	0.55
N4	Refugial	N	1363	18.5	−2.8	2187	35	391.9002	69.2	0.44
S1	Expanded	S	895	25.2	−0.5	3531	16	620.3911	167.3	−0.26
S5	Refugial	S	1517	19.4	−3.8	2162	52	421.0679	18.7	0.54

Tmin, Tmax = average minimum, maximum temperature, sum GDD = sum of the number of days with temperature >5.5 °C, NFD = Number of frost days, number of days with temperature <4 °C, VPD = vapour pressure deficit (the difference between the actual amount of air moisture and the amount of air moisture at saturation), stress May to September = difference between evapotranspiration and precipitations, scPDSI_ self-calibrated Palmer Drought severity index (Water stress between May and September (stress) was calculated as the difference between ETP and precipitation, and the self-calibrated Palmer Drought Severity Index (scPDSI) was calculated using the scPDSI R package (Wells et al. 2004). See Davi and Cailleret (2017) for raw data.

## Material and methods

### Species, study site, and sampling

*Fagus sylvatica* L., the European common beech, is a dominant broadleaved tree species of many lowland and mountain forests across Europe, extending from Spain to the Carpathians and from Sicily to southern Sweden. Its genome is diploid with 2n = 24 chromosomes. It has a monoecious, anemophilous, and mainly allogamous mating system (Vornam et al. 2004). Sexual maturity is reached at 40–50 years of age. Seeds are mostly produced in mast years and are mainly dispersed at short distances (mean primary dispersal distance of 25 m, Bontemps et al. 2013), but non-negligible long-distance dispersal can be achieved (typically with birds such as the European jay, *Garrrulus glandarius* (Nilsson 1985).

The population we have studied on Mont Ventoux is one of the 52 glacial refugia identified by Médail and Diadema (2009) in the Mediterranean basin. Mont Ventoux is a 1920 m-high mountain located in southern France, with a steeper, colder northern slope and a flatter, warmer southern slope. Both harbour several floristic altitudinal zones encompassing a wide range of ecosystems, from Mediterranean to alpine, with beech occurring in most of them (Barbero et al. 1978), on both slopes.

In historical times, beech populations have been restricted to a relict area on the top of both the north and the south slope, with an extent that shrank progressively until reaching its minimum in the mid-19th century. The paleoanthracological survey of Gamba et al. (2023) reports the retrieval of remnants of beech charcoal at 1130 m of altitude on the northern slope and dated them to 346–460 years before present (1490–1604 CE). Since 1850, beech stands expanded continuously until almost doubling the surface they occupied, reaching in the process elevations of 300–600 m below the lower margin of the historical relict areas. A previous investigation of demographic scenarios using coalescent-based simulations and microsatellite genetic data revealed the genetic signature of population expansion, but not that of bottlenecks (Lander et al. 2011), with the total “ancestral” (i.e., refugial) effective population size estimated to approximately 1000 to 13,000 individuals (Lander et al. 2011). Weak but significant divergence among different portions of the historical relict population was detected, showing that, in spite of the close proximity of the forest patches, gene flow was somewhat restricted, allowing sub-populations to differentiate (mean divergence of each subpopulation from the global population β_WT_ = 0.03; Lander et al. 2011). On the contrary, no sign of differentiation was detected between recently expanded forests and the relicts they expanded from (Lander et al. 2021), suggesting that the time elapsed since the start of the expansion is insufficient for neutral divergence processes to leave genome-wide marks. Therefore, the beech stands of Mont Ventoux show signs of recent, ecologically-driven divergence (Csilléry et al. 2014; Gauzere et al. 2020), in the absence of neutral divergence (Lander et al. 2021), which enables the detection of even relatively weak signals of adaptive genomic divergence.

To carry out this study, we sampled 20 to 26 adult trees from four stands identified in Lander et al. (2021): N3 and N4 on the northern slope and S1 and S5 on the southern slope (see Table 1 for details on elevation and environmental parameters). N3 and N4 belong to a continuous mixed forest of beech and silver fir (*Abies alba* Mill.) which extends over a steep altitudinal gradient, and are close to each other, even though they are separated by 150 m in elevation, and thus have a difference in mean temperature of about 2 °C. The S5 stand grows at the highest elevation, and is a part of a continuous beech forest encompassing the mountain ridge. Its mean annual temperature is intermediate between N3 and N4, but it experiences colder winter temperatures. Summer vapour pressure deficit is similar among N3, N4, and S5, but the latter is globally the least exposed to drought (Table 1, stress May to September). S1 is a mixed stand of beech and oak (*Quercus* spp. L.) located on a small gorge within the lowest part of the south-eastern mountainside, and it is the population at the lowest elevation, experiencing the highest mean temperatures and the most severe drought stress (Table 1, stress May to September and self-calibrated Palmer Drought Severity Index, scPDSI). According to Lander et al. (2011; 2021), stands N3, N4, and S5 belong to the historical relict area, while stand S1 is the result of the demographic expansion process that occurred between the nineteenth and the twentieth centuries, likely originating in the upper south slope to which S5 belongs. In the Supplementary materials of Lander et al. (2021), N3, N4, S1, and S5 correspond respectively to W_N3, W_384, S_1, and S_5.

### Genotyping by sequencing

The genotypes were obtained based on data from a sequence capture experiment following Peñalba et al. (2014). The sequence capture was carried out by RapidGenomics (Gainesville (FL), US) with the probes reported in Supplementary Table ST1. The probes were based on a beech unigene set (Lesur et al. 2015), and therefore the targets were exclusively coding sequences. Probe preparation, fragment capture, sequencing, de novo assembly, read mapping, as well as variant calling and filtering and the preparation of genotype tables, were executed as described in Scotti et al. (2023; the whole process was actually carried out concurrently with the treatment reported there). In summary, reads were de novo assembled using the velvet algorithm (Zerbino 2010) with K-mers (that is, the substrings used to identify alignments; see Compeau et al. 2011) of length 85 nucleotides, coverage cut-off = 8, minimum contig length = 100 and expected coverage = 20. Because assembly algorithms are very sensitive to polymorphism, that prevents contigs from being correctly assembled, we carried out de novo assembly only from one individual (Sample Fs_T_S1_005), chosen because it had the largest number of high-quality reads.

Notice that the data were obtained and curated before the beech genome reference (Mishra et al. 2022) was obtained and published, and therefore, at the time of bioinformatics data treatment, de novo assembly was the best available strategy. The de novo contigs were mapped to the reference genome (Mishra et al. 2022) for further curation, insight and annotation, using BLAST 2.9.0+ (Altschul et al. 1990; Zhang et al. 2000). To obtain the positions of target regions and SNPs on the reference genome, we proceeded as follows. We first extracted the best hit for each target region and retrieved the positions of each nucleotide in the target regions relative to the reference scaffolds (including gaps in the alignment). Pairs or small groups of contigs mapping to the same genomic regions, or with no hits, were removed from the dataset. We then built a correspondence table providing the position of each nucleotide of each target region on reference scaffolds. Using this table, target region positions of SNPs were converted into scaffold positions, which were used in all subsequent analyses. For analyses at the target region level, the position of each target region on the reference genome was defined as the start of its alignment on the scaffolds. The random distribution of target regions along chromosomes was also tested using a test for conformity to a Poisson distribution (see Supplementary Methods SM1 for details).

Based on the reference contigs obtained above, we performed read mapping with BOWTIE2 v 2.0.6 (Langmead and Salzberg 2012) using “very- fast-local” parameters; we used SAMTOOLS MPILEUP (Li et al. 2009) to build a mpileup file with all the individual alignments to the reference The parameters applied for variant calling (using VARSCAN v. 2.3.9, Koboldt et al. 2009) were the following: minimum coverage = 20, minimum average quality = 30, minimum variant frequency = 0.3, minimum frequency for homozygote call = 0.7, maximum missing data = 20%. We further filtered the dataset according to the distribution and the biological properties of the variant were used to further filter the dataset with the ad hoc R (R Core Team 2023) *sieve()* script. The *sieve()* script works on a VCF file and filters out contigs based on SNP density (variants per base) and heterozygosity thresholds; it also returns genotype tables. We excluded the contigs with variant density > 0.05 (i.e., having more than one variant every 20 bases; *varDensThreshold* argument) and carrying only heterozygous genotypes to remove contigs potentially containing paralogues (*hetThreshold* argument = 1). The filtering process was refined by removing monomorphic sites with the ad-hoc *monoRemove()* R script.

### Diversity and Differentiation Statistics

We carried out diversity and differentiation analyses in R v. 4.1.2 (R Core Team 2023), unless stated otherwise. The values of π per base were computed for each population as the average *π* over all nucleotides of that sequence, including the invariant ones. Global *F*_ST_, following Weir and Cockerham (1984), and its variance components relative to stand and slope, were computed with the function *varcomp.glob()* from the package Hierfstat 0.5–7 (Goudet 2005). We assessed the 95% confidence intervals for the global statistics by bootstrapping 1000 times the loci with *boot.vc()*. We also estimated *F*_ST_ values for each stand pair with the function *pairwise.WCfst()*. The hypothesis of non-null genetic differentiation was tested by bootstrapping over loci 1000 times with *boot.ppfst()*, and checking significance both at the 95^th^ and 99^th^ quantile. We obtained an UPGMA clustering tree from pairwise *F*_ST_ distances using Phangorn 2.5.5 (Schliep 2011).

Because *F*_ST_ estimates of divergence are sensitive to within-population diversity (Jost 2007; Lefèvre and Gallais 2020) we also computed pairwise estimates of Jost’s D (Jost 2008), which is unaffected by such biases. We tested the hypothesis of non-null genetic differentiation by bootstrapping over loci 1000 times and assessed the significance at the 0.95 confidence interval. See Data availability section for the scripts (n°4–10).

### Demographic model and simulation of genotypic data

**Fig. 2:**
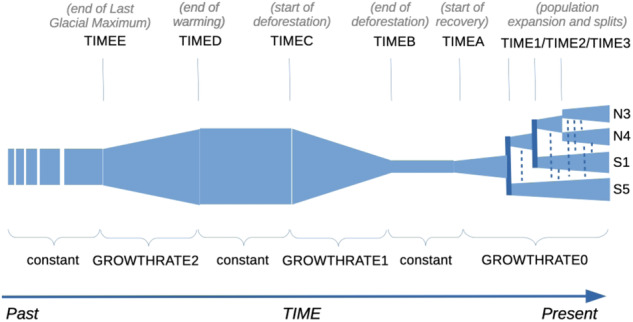
Graphical illustration of the events that were modelled in the background demographic model. The time arrow is depicted horizontally, with present time to the right (arrow at the bottom of the plot). The names of parameters estimated in the demographic inference are shown in bold capitals: TIME parameters are shown above the diagram (italic texts in parentheses describe the geological and historical events corresponding to each time point), and GROWTHRATE (population growth) parameters are shown below the diagram. The filled polygons indicate population sizes (POPSIZE) through time (with thicker vertical width indicating larger population size). Towards the present, the ancestral population splits progressively into the four current (sub)populations that have been studied in the paper (N3, N4, S1, S5). Dotted vertical lines indicate continued gene flow among all population pairs. The broken box at the left end indicates that the population was considered as constant over a long and undefined time in the past. Polygon sizes, lengths, and shapes are entirely arbitrary and are not to scale with either the starting range or the maximum likelihood estimations of the demographic parameters; (b) the topology of population splits is shown only for illustration, as the exact timing of each event was left for the inference algorithm to estimate.

We built on the previous work of Lander et al. (2011), which allowed the major demographic events to be identified and the parameter range to be set. We adapted their demographic model to our sampling design with four stands as follows and as illustrated in Fig. 2. We used the demographic parameters they estimated to inform the starting parameter ranges for maximum-likelihood estimation. Lander et al. (2011, 2021) also report that the Mont Ventoux beech stands reached their minimum extent in the 1850s, while Gamba et al. (2023) report beech charcoal in pits of the northern slope of Mont Ventoux from 925 to 1340 m a.s.l. and dated most of them not earlier than the Middle Ages. They also dated beech charcoal found at 1130 m a.s.l. to the Modern Era, confirming the existence of beech trees on the northern slope before the reforestation programme started in the 1850s. A continental-scale palynological survey by Magri (2008) observed that, after the Würm glaciation, beech naturally expanded from its glacial refugia. However she noted that climate and anthropogenic pressures may have locally affected its colonisation patterns. Martin-Gousset et al. (2019) provided further information on the dynamic of the post-Neolithic deforestation dynamic on the Mont Ventoux. These dates allowed us to define starting parameter ranges for historical expansion and contraction dates. We relied on these pieces of information and the divergence estimates obtained from our genomic data (see above and Results section) to build our scenario, which expands from the event times and population sizes, estimated by Lander et al. (2011, 2021), further back in the past until the Holocene expansion: we started with an expansion (simulating the Holocene colonisation following climate warming; TIMEE to TIMED, GROWTHRATE2), followed by a time of stability (TIMED to TIME C) and contraction (matching the progressive deforestation since the Neolithic; TIMEC to TIMEB, GROWTHRATE1), until reaching the minimum-extent size, followed again by stability (TIMEB to TIMEA); and finally, we allowed the four stands to expand again and diverge starting from the minimum-extent population, while exchanging migrants (TIMEA to TIME3, GROWTHRATE0), until present time. The demographic model included 21 parameters in total (Supplementary Table ST3).

To identify the most-likely values for demographic parameters, and to simulate genetic data matching the estimated demographic history of the populations, while taking into account uncertainties in model parameter estimation (Myers et al. 2008; Lapierre et al. 2017), we used the following three-steps strategy (see Supplementary Methods SM2 for details):

(a) the first step consisted in estimating the 21 parameters from empirical data. To do this, we used the Maximum-Composite-Likelihood estimation tool implemented in FastSimCoal2 v2.7.0.5 (Excoffier and Foll 2011), based on the folded pairwise site-frequency spectra (2D-fSFS) for each population pair (see Supplementary Methods SM2 for details on how the 2D-fSFS were obtained from genotype data). Note that the FastSimCoal2 optimisation algorithm is allowed to sample parameter values outside the boundaries set by the user, so the maximum likelihood estimations of the parameters (Table 2) do not necessarily reflect their starting ranges (see Supplementary Methods SM2). We performed 100 runs to obtain a distribution of values for each parameter;

**Table 2:** Summary statistics of the 100 independent estimates for each of the 21 demographic simulation parameter.

	Description	lower CI boundary	median	mode	upper CI boundary
POPSIZE-N3	Current size of population N3	601	23383	18521	37882
POPSIZE-N4	Current size of population N4	42482	44528	46345	46573
POPSIZE-S1	Current size of population S1	652	23729	30362	38128
POPSIZE-S5	Current size of population S5	304	20242	29609	33290
MIG N3-N4	Symmetrical migration rate for all population pairs (independent draws for each population pair)	0.0013	0.0022	0.0021	0.0057
MIG N3-S1		0.0014	0.0026	0.0043	0.0070
MIG N3-S5		0.0013	0.0024	0.0028	0.0067
MIG N4-S1		0.0013	0.0022	0.0015	0.0056
MIG N4-S5		0.0013	0.0022	0.0035	0.0058
MIG S1-S5		0.0012	0.0023	0.0062	0.0061
TIME1	Time of N3-N4 merger	1	1	1	3
TIME2	Time of S1-(N3-N4) merger	1	1	1	3
TIME3	Time of final population merger (S5-(S1-(N3-N4)))	1	1	1	5
TIMEA	End of population contraction	2	2	2	12
GROWTHRATE0	Growth rate until TIMEA	0.00443	0.00313	0.00366	−0.00010
TIMEB	End of population stability at minimum size	2	4	3	16
TIMEC	End of population expansion	90	98	99	99
GROWTHRATE1	Growth rate until TIMEC	−0.00010	−0.00127	−0.00125	−0.00544
TIMED	Start of population contraction	101	159	103	268
GROWTHRATE2	Growth rate until TIMEE	0.00001	−0.00001	−0.00004	−0.00004
TIMEE	End of population contraction	176	937	987	989

See the text and Supplementary Table ST3 for the description of simulation parameters. The mode corresponds to the peak to the density distribution of the parameter (see Supplementary Material).

(b) the second step consisted of sampling meaningful combinations of correlated parameter values obtained in (a). Since several parameters were correlated partly by construction, because of their boundaries were defined by other parameters, we sampled them together to avoid ending up with biologically implausible combinations of parameter values as input for simulations. Hence, we analysed the correlation among parameters and identified “correlation blocks”. We then selected a “core” parameter in each block (i.e., the one with the strongest correlation with all others in the block) and drew 100 values for this core parameter from a uniform distribution, with boundaries set to the minimum and maximum of the distribution obtained in (a). The values of the remaining parameters were computed by performing linear regressions between them and the from value of the “core” parameters values (including error). This allowed us to generate 100 new sets of values for the 21 parameters.

(c) In the third step, these 100 sets of parameter values were used as input for FastSimCoal2 to simulate genotypes for four populations, which we could use as the demographic-neutral reference in the following outlier detection analyses (see below for details). In each simulation (each starting from one set of parameter values), we simulated 750 1000-bp DNA independent fragments, with per-base recombination rates obtained from the comparison of the linkage map distances and physical genome distances reported in Mishra et al. (2022); we used the same mutation rate of 9.5 × 10^−10^ reported in Milesi et al. (2023). This way we obtained 100 genome-level 4-population SNP data sets, which were compiled together to obtain a single data set (“*simulated data set*” from here on) with composite diversity properties, representing the properties of data obtained from all sets of parameter values.

### Detection of divergence outlier loci between stand pairs

We applied three different methods (see below) to identify genomic loci that show unusual allele frequency differentiation among stands (divergence outlier loci in the following). For each method, we followed the same approach to account explicitly for population demography, as described below.

We first applied the method on the *simulated data set*, to generate the neutral, demography-driven *simulated distribution* of the method-specific divergence statistic for each population pair. Then, we applied the method on the empirical loci, thus obtaining single-locus values of the divergence statistic and an *empirical distribution* of the divergence statistic.

To identify divergence outliers, we compared the value of the divergence statistic obtained for each empirical locus and stand pair to the corresponding *simulated distribution*. This allowed us to compute a P-value for each empirical value of the statistic as the normalised rank *r*_*i,p*_ of the value of the statistic for locus *i* for stand pair *p* relative to the simulated distribution for stand pair *p*. The P-value *P*_*i,p*_ was computed as *P*_*i,p*_ = *r*_*i,p*_ / N, with N = total number of loci in the simulated distribution. *P*-values were converted into *q*-values using the R package “*q*value” (Storey et al. 2023). An FDR = 0.01 was used as a threshold to identify outliers.

**Fig. 3:**
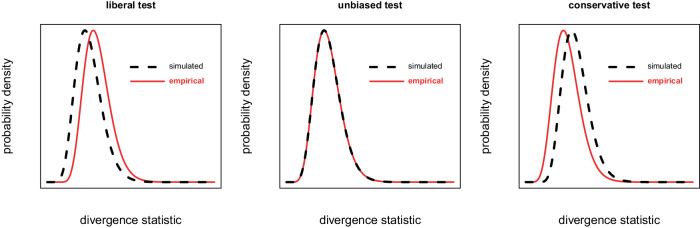
Illustration of the biases in tests for the search of divergence outliers. Filled line: distribution of *empirical* values of the divergence statistic; dashed line: distribution of the *simulated* values of the divergence statistic. Liberal test: the simulated distribution is shifted to the left relative to the empirical distribution; it is easier for empirical values to have extreme values relative to the simulated values they are compared to, and this generates false positives; unbiased test: the simulated distribution is centred on the empirical distribution; this should generate little false negatives or positives; conservative test: the simulated distribution is shifted to the right relative to the empirical distribution; it is more complicated for empirical values to have extreme values relative to the simulated values they are compared to, and this generates false negatives.

The comparison of the *empirical* and *simulated* distributions of the divergence statistic allowed us to assess whether the outlier tests of our methods were liberal, unbiased, or conservative (Fig. 3): when the *simulated* distribution turned out to be shifted to the *left* relative to the *empirical* distribution, we considered the test as liberal (i.e., too many empirical values exceeded the threshold built on the simulated distribution); when the *simulated* distribution turned out to be shifted to the *right* relative to the *empirical* distribution, we considered the test as conservative (i.e., too few empirical values exceeded the threshold built on the simulated distribution); when there was no difference between the distributions, we considered the tests as unbiased. A two-sided Wilcoxon test was used to assess the shift between distributions.

We tested signatures of selection with three methods resting on different assumptions, with null expectations obtained, for each of them, based on the aforementioned demographic simulations:

(1) The G2D statistic (Nielsen et al. 2009) is based on the calculation of the multinomial composite likelihood ratio (CLR) of observing a given combination of allele frequencies, at the SNPs carried by a given stretch of DNA sequence, in a pair of stands, relative to the expectation obtained by genome-wide allele frequencies. It therefore applies to DNA sequences carrying at least two SNPs and uses 2D-fSFS as a starting point. G2D was computed using the equations for the G statistic as in Nielsen et al. (2005), as modified to apply to a 2D-fSFS. The analyses were carried out at the contig level (see the script in°18 on the online repository for details of the calculation of the G2D statistic).

(2) For each stand pair and for each locus, the absolute difference in allele frequency was computed between stands, and then the distribution of allele frequency differences was obtained for all SNPs for a given stand pair, both for empirical and simulated data.

(3) Bamova (Gompert and Buerkle 2011) estimates components of molecular variance, genome-wide and for individual loci. To run our analyses, we extracted the modes of locus-specific posterior distributions of *Φ*_ST_ as the divergence statistic, and proceeded as described above to identify outliers. See Supplementary Methods SM3 for details.

Notice that the first method (G2D) assesses divergence at the level of a whole sequence (here, a target region), while the other methods assess divergence at the single SNP level.

#### “Standard” (non-demography controlled) Bamova outlier search

For comparison, we also applied the “standard” Bamova (Gompert and Buerkle 2011; Scotti et al. 2023) method to the raw data (that is, without accounting for population demography). Note that G2D and allele frequency difference tests can only be run with a demographic model, and that allele frequency difference (AFD) has no theoretical expectation and therefore no parametric test could be applied to it. Instead, Bamova relies on a theoretical island population model as a null model, so we were able to test the methods under their theoretical assumptions, following Scotti et al. (2023). Outlier loci were identified based on the Bayes Factor statistics (Makowski et al. 2019), the difference between the value of the prior and the posterior densities at x – that is, the ratio of the data under the null hypothesis and the alternative hypothesis. Hence, results are drawn directly from the observed data.

### Annotation of SNPs and genes

We used SnpEff 4.3 (Cingolani et al. 2012) to functionally annotate each outlier SNP and target region relative to the reference genome annotation (Mishra et al. 2022) (see Supplementary Methods SM4). We retrieved the gene-level functional annotation from the GFF3 annotation reported in Mishra et al. (2022) and matched with orthologs with the same accession on GenBank.

To search for correspondences with published studies, the genomic regions containing outliers were compared, using Blast (or manually, when locus positions relative to Mishra et al. (2022) were reported), to outlier sequences reported in Müller (2017; drought response expression outliers), Pfenninger et al. (2021; association to drought damage), Csilléry et al. (2014; altitudinal divergence in the same populations as in the present study), Lesur et al. (2015; differential expression during budburst), Postolache et al. (2021; rangewide signals of adaptive divergence), Meger et al. (2021; adaptive divergence for phenology).

## Results

The de novo assembly resulted in 11,527 contigs, amounting to 4,407,713 bp. Variant calling produced 40,594 variants in 9195 genomic regions. After filtering, the dataset included 35,997 SNP in 8970 genomic regions (3,487,133 bp), with 2.9% missing genotypes. After mapping the contig sequences onto the beech reference genome, only contigs with a unique position on the reference were retained, resulting in a VCF file containing 34,889 SNPs from 8791 target regions mapped unequivocally to as many scaffold positions (amounting to 3,429,878 bp, or 0.6% of total genome length), and 2.8% missing data. Target regions were not randomly distributed along chromosomes (see Supplementary Results SR1).

After subsetting the dataset into stand pairs, the number of polymorphisms for each stand pair ranged between 30,966 (N3 - S1, 45 individuals) and 32,033 (N4 - S5, 51 individuals) (see Supplementary Table ST2). This reduction of number of polymorphisms is, of course, caused by the removal of the variants only carried by the samples excluded from each subset.

### Diversity and Differentiation Statistics

**Fig. 4:**
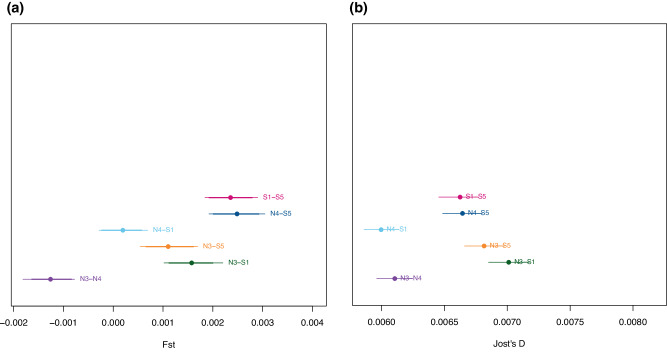
Levels of pairwise molecular divergence for all population pairs, computed as an average of divergence over all SNP. Filled dots indicate the empirical (observed) values, and horizontal bars indicate 95% confidence intervals, as obtained by bootstrap. **a** *F*_ST_; (**b**) Jost’s *D*.

We computed diversity statistics for each stand as well as global and pairwise values of differentiation statistics. The values of normalised diversity $\pi$, averaged over genomic regions, were 2.089 × 10^−3^ for N3, 2.121 × 10^−3^ for N4, 2.122 × 10^−3^ for S1, and 2.215 × 10^−3^ for S5. Global *F*_ST_ was 0.0010 (95% confidence interval (CI): 0.0008–0.0013, median 0.0011). Pairwise *F*_ST-_values ranged between −0.0013 and 0.0025, with a mean value of 0.00130 (setting negative values as 0) (median = 0.0013) (Fig. 4a). The N4-S5 plot pair displayed the largest differentiation (median = 0.0025), and the N3-N4 the smallest (medians −0.0013; the 99% CI for N3-N4 was entirely negative). Pairwise Jost’s D values ranged between 0.0060 (N4-S1) and 0.0070 (N3-S1) (mean = 0.0065, median = 0.0066). No confidence interval on Jost’s D-values encompassed zero (Fig. 4b). Most confidence intervals for pairwise divergence overlapped at least with the confidence interval of another population pair, making it difficult to establish a clear dendrogram of population split, although globally, N3 and N4 seem to form a pair, and this pair seems to be more tightly related to S1 than to S5. We used the topology; (S5-(S1-(N3-N4))) as a starting point for the demographic analyses, but left the algorithm free to establish the order of population separation.

### Demographic model and simulation of genotypic data

After having computed diversity statistics, we moved to computing the maximum-likelihood estimates of the demographic parameters. The mode and median of estimated current population size (Table 2) were an order of magnitude larger than the upper boundary of the parameter starting range (Supplementary Table ST3). Population sizes were in the order of few tens of thousands for all stands, with the confidence interval on N4 stand size much narrower than the other stands’ (Table 2). Splitting of populations was estimated to have occurred not earlier than five generations ago (a timeframe that corresponds to the upper boundaries of the estimates of TIME1, TIME2, and TIME3; Table 2, Fig. 2). This suggests a very recent expansion from historical relict areas, which started at the earliest twelve generations ago (upper boundary for TIMEA; Table 2, Fig. 2), with relatively small growth rates of effective (not census) population sizes. In fact, the estimates of GROWTHRATE0 (Table 2, Fig. 2) were between 0.00010 and 0.00443, with a mode (0.00366) closer to the upper boundary, thus supporting *expansion*). However, the exact order of population separation remained unresolved, as the intervals for all population split times overlapped. Contraction (possibly due to deforestation in historical times) ended at the earliest 16 generations ago (upper boundary for TIMEB; Table 2, Fig. 2), and started in the order of one hundred generations ago (TIMEC; Table 2, Fig. 2); the model correctly returned negative values (i.e., *contraction*) for GROWTHRATE1 (−0.00010/0.00544, Table 2, Fig. 2). Deep-time population expansion is estimated to have occurred between 100–300 and 1,000 generations ago (TIMED, TIMEE; Table 2, Fig. 2), but these estimates are not very precise. Growth rates would have been very small, with estimates around zero, and mostly with negative values, suggesting *contraction* instead of expansion of effective population sizes. The distribution of the maximum-likelihood estimates of the demographic parameters are reported in Supplementary Methods 2, Supplementary Figure SM2-2.

### Detection of divergence outlier loci between stand pairs

Based on the demographic background established through the empirical simulation we could move to the identification of the outlier loci in the empirical data. Globally, we detected 27 signatures of selection from 19 non-overlapping genomic regions (Table 3). All the six tests of the G2D method were unbiased (Supplementary Table ST4). Seventeen target regions showed significant divergence in at least one stand pair at the 0.01 FDR; one of them (Bhaga_8.g1884) showed divergence for two stand pairs, and one (Bhaga_7.g1751) for three stand pairs. Populations pair showed variable numbers of outlier target regions (N3-N4: three; N3-S1: two; N3-S5: four; N4-S1: four; N4-S5: five; and S1-S5: one).

**Table 3:** List of outliers.

Gene number	chr	Region	Gene description	Divergence tests							Non-demographic outlier
				G2D		AFD		Bamova		Ann	
				pair	POS	pair	POS	pair	POS		
Bhaga_1.g6628	1	66574103 - 66580231 (+)	**• XP_023915805.1** **•** glycerol-3-phosphate dehydrogenase [NAD(+)] GPDHC1, cytosolic **•** Energy production and conversion			**S1-S5**	**66575559**	**S1-S5**	**66575559**	I,mo	S1-S5
Bhaga_2.g4572	2	41650033 - 41655512 (−)	**• KAB1208090.1** **•** Glucan endo-1,3-beta-glucosidase 2 **•** Possibly involved in carbohydrate binding	N4-S1	41651123- 41651459						
Bhaga_2.g4634	2	42363076 - 42376634 (−)	**• XP_023922067.1** **•** tRNA threonylcarbamoyladenosine dehydratase **•** Activating enzymes (E1) of ubiquitin-like proteins	N3-S5	42370573- 42370965						
Bhaga_4.g1667	4	14159632 - 14169373 (+)	**• XP_030964976.1** **•** (unknown gene name) **•** probable disease resistance protein RF9			**S1-S5**	**14168662**	**S1-S5**	**14168662**	M,md	S1-S5, N4-S1,
Bhaga_4.g1705	4	14494635 - 14499931 (−)	**• XP_023889455.1** **•** isoflavone reductase homologue **•** oxidoreductase activity, acting on the CH-CH group of donors, NAD or NADP as acceptors	N3-S5	14494838- 14495102						
Bhaga_5.g1593	5	14195661 - 14197196 (+)	**• PON33942.1** **•** (unknown gene name) **•** NTF2-like domain containing protein	N3-S5	14195973- 14196472						
Bhaga_5.g4748	5	40781598 - 40785294 (+)	**• XP_023913931.1** **•** adenylyltransferase and sulfurtransferase MOCS3 **•** Cofactor biosynthesis: molybdopterin biosynthesis. tRNA modification: 5-methoxycarbonylmethyl-2-thiouridine-tRNA biosynthesis	**N4-S1**	**40781565-** **40781959**			**N4-S1**	**40781880**	S,lo	N4-S1, N3-S1
Bhaga_6.g2513	6	22741089 - 22752585 (−)	**• RVW32758.1** **•** Trasposon TX1 uncharacterised 149 kDa protein **•** Non-LTR retrotransposon and non-LTR retrovirus reverse transcriptase (RT) which catalysers the conversion of single-stranded RNA into a single-stranded DNA	N4-S5	22743486- 22743973						
Bhaga_7	7	-	**• KU700965.1** **•** putative LOV domain-containing protein^a^ **•** sensors for light and oxygen in signal transduction	**N4-S5**	**3608009-** **3608280**			**N4-S5**	**3608194**		N4-S5
								**N4-S5**	**3608089**		N4-S5
Bhaga_7.g1751	7	14815926 - 14822900 (+)	**• XP_023905227.1** **•** TIP41-like protein **•** Regulation of phosphoprotein phosphatase activity and TOR signalling; could play a role in cytoskeleton functions.	N3-N4 N4-S1 N4-S5	14822493- 14822895						
Bhaga_7.g4066	7	35377853 - 35383008 (+)	**• XP_030971157.1** **•** putative PAP-specific phosphatase, mitochondria **•** catalyses the hydrolysis of 3’-phosphoadenosine-5’-phosphate (PAP) to AMP	N3-S5	35385076- 35385440						
Bhaga_8.g97	8	774878 - 784334 (−)	**• XP_030974634.1** **•** exosome complex exonuclease RRP44 homologue A **•** rRNA processing and catabolic process; RNA and metal ion binding; 3’-5’ RNA exonuclease activity into RNase complex (nucleus and cytosol)	N3-S1	773129- 773569						
Bhaga_8.g1884	8	15669815 - 15678133 (−)	**• XP_024017681.1** **•** CMP-sialic acid transporter **•** pyrimidine nucleotide-sugar transmembrane and sialic acid transporter activity (Golgi)	N3-N4 N4-S5	15673749- 15674000						
Bhaga_9.g1595	9	12879937 - 12884370 (−)	**• KAB1210082.1** **•** (unknown gene name) **•** Dynein assembly factor 1, axonemal	N4-S5	12881196- 12881598						
Bhaga_9.g2261	9	18401049 - 18401988 (+)	**• KAB1227406.1** **•** putative vacuolar protein sorting-associated protein 13 C	N3-S1	18401752- 18402280						
Bhaga_10	10	-	**• XM_024042142.1** **•** (unknown gene name) **•** mitochondrial transcription termination factor family isoform 1^a^	N4-S1	10719018- 10719314						
Bhaga_10.g2539	10	20777894 - 20784927 (−)	**• VVA22558.1** **•** (unknown gene name) **•** uncharacterised B456_004G223300	S1-S5	20781693- 20782152						
Bhaga_10.g3505	10	28867540 - 28869330 (−)	**• XP_023906757.1** **•** (unknown gene name) **•** NAC domain-containing protein 87-like	N4-S5	28868621- 28868946						
Bhaga_11.g539	11	4738139 4743880 (+)	**• KAA3460747.1** **•** BRO1 domain-containing protein BROX **•** Metallo-hydrolase/oxidoreductase	N3-N4	4738984- 4739355						

The position on the reference genome, the annotation of the corresponding genomic region, and the stand pairs for which the outlier was identified are reported. When sequence-level (G2D) and/or nucleotide-level (Allele Frequency Difference (AFD), Bamova) outliers belong to the same region, they are displayed on the same row and their stand pair and position are highlighted in bold. All genes were annotated using the gff3 provided by Mishra et al. (2022) and GeneBank, unless indicated otherwise.

Gene number = gene number reported in the Mishra et al. (2022) annotation, chr = chromosome number (Mishra et al. 2022), Region = genomic coordinates of the target sequence in bases pairs, Gene description = GenBank accession number of the most similar DNA sequence and summary description of function, pair = stand pair for which the locus was an outlier, POS **=** position on the chromosome, Ann = functional annotation of SNP, I = intron variant, M = missense, S = synonymous, mo = modifier, md = moderate, lo = low.

^a^annotation derived from orthologous genes identified by a BLAST search, followed by a match for the accession on GeneBank.

Allele frequency difference tests were either unbiased (for the N4-S5 and S1-S5 pairs) or conservative (for the four remaining pairs) (Supplementary Table ST4). Allele frequency differences exceeded the neutral expectation at two loci (Bhaga_1_66575559 and Bhaga_4_14168662) for the S1-S5 pair (See Table 3).

All the Bamova tests were conservative (Supplementary Table ST4). We retrieved five outliers: one in N4-S1 (Bhaga_5_40781880), two in N4-S5 (Bhaga_7_3608194 and Bhaga_7_3608089), and two in S1-S5 (Bhaga_1_66575559 and Bhaga_4_14168662).

The two AFD outliers were retrieved also by Bamova, and several Bamova outliers were inside an outlier target region identified by G2D (Table 3 and Fig. 4), while fifteen out of the seventeen G2D outliers were unique. With the “standard” approach, many more outliers were found with BAMOVA: 65 between populations N3 and N4; 120 between S1 and S5; 88 between N3 and S5; 109 for N4 and S5; 79 for N3-S1; and 74 for N4-S1 (Supplementary Result SR1, Supplementary Table 5).

Outlier gene and SNP annotations (gene functions and substitution effects) are reported in Table 3. All outlier-containing genomic regions matched at least one sequence in the transcriptomic or genomic regions analysed in Müller et al. (2017), Pfenninger et al. (2021), and Lesur et al. (2015), but none of our outliers appeared in the outlier lists reported in those studies. Our outliers did not match any of the candidate genes analysed in Postolache et al. (2021), Meger et al. (2021), or Csilléry et al. (2014). Some of the outliers found with the “standard” approach overlapped with those found in the cited studies (Supplementary Result SR1, Supplementary Table 5).

## Discussion

Using a combination of high-throughput genomic data and demo-genetic modelling tools, we found molecular signatures of microgeographic adaptation in a European beech population encompassing the two slopes of a Mediterranean mountain. Although the studied stands have been separated only by a few generations, this genomic signal of divergent selection confirms that population recolonisation and the occupation of novel environments were followed by selective processes strong enough to provoke population divergence. The demographically explicit approach used here, based on a combination of historical records with the results previously obtained by Lander et al. (2011), opens new perspectives for understanding the genomic architecture of microgeographic selection. After controlling for demographic effects, 27 signatures of divergent selection were detected (in 19 out of 8791 sequenced genomic regions): 20 in G2D, two as allele frequency differences (AFD), and five in BAMOVA (Table 3). A subset of divergence outliers showed a consistent signal over stand pairs and/or methods: out of 19 outlier genomic regions, four appeared with two methods, and two for at least two stand pairs (Table 3, Fig. 4). This suggests that signals of divergent selection are strong enough to be picked up by multiple methods and that, for at least some loci, similar selective processes occur within multiple stand pairs, because of shared environmental contrasts (Table 1).

We estimate 0.19% of the genome (sequence level; G2D method) and 0.1% of the sequenced genomic polymorphisms (Bamova, AFD) to be under selection. This reflects the findings of Kelly (2022), who found a comparable proportion of genomic loci responsive to temporal selection in a longitudinal study spanning 23 generations in monkeyflower (0.1%, 994 SNPs the difference in frequency of which fluctuated significantly from positive to negative, harboured in 1000 genes), but also the findings on several other forest tree populations: Mosca et al. (2016) (0.16% among 768 SNPs from *Pinus cembra* and 1152 SNPs from *Pinus mugo*); Brousseau et al. (2021) (0.19% among 97,062 SNPs in *Eperua falcata* Aubl.); and Scotti et al. (2023) (0.1 - 1.0% on panels of 7114 - 8462 SNPs in four conifer species: *Abies alba*, *Cedrus atlantica*, *Pinus halepensis*, and *Pinus pinaster*). Microgeographic adaptation apparently involves a small fraction of the genome, even though Scotti et al. (2023) hypothesised that a fraction of truly adaptive loci went undetected because of the lack of power. Because our tests were often conservative, it is possible that we also missed a fraction of the true divergence outliers.

Population-genetic theory predicts that fewer, larger-effect loci should underlie *local* adaptation relative to *global* adaptation (Yeaman 2022). Therefore, it is not surprising that we identified few outliers, seemingly undergoing strong selection, even though a compact genetic control should limit adaptation to very divergent local conditions (Cubry et al. 2022). It is worth noticing that large-effect mutations that confer advantage in new environments can actually favour population expansion (Gilbert and Whitlock 2017); such loci could therefore actually have facilitated recolonisation.

A clear (methodological) message to be received from the small numbers of outliers found here comes from the comparison with the analyses carried out without demographic correction: taking into account demography greatly reduces the number of positive signals in the Bamova analyses, probably by removing large numbers of false positives. The comparison, however, is imperfect, because the method not controlling for demography (Scotti et al. 2023) uses Bayes Factors (computed from empirical values only) instead of q-values (computed by comparison with simulated values) to identify divergence outliers, and because our tests were often conservative (that is: divergence levels obtained based on the demographic model were on average higher than the empirical divergence levels), producing some amount of false negatives (that is, even high empirical divergence levels fell within the middle range of the simulated values). These limitations notwithstanding, this comparison suggests that incorporating historical information in demographic models, and then using them as a background for the analysis of adaptation, should become standard practice whenever possible. This will allow tests to be performed without relying on the equilibrium hypotheses that often underlie the neutral expectations used to identify outliers. In general, it’s always better to rely on diverse methods to detect divergence outliers according to the available knowledge on the studied population: methods resting on different assumptions can capture different aspects of the adaptive process. Whenever linkage disequilibrium is strong between neighbouring SNPs, a multilocus method such as G2D should be more fitting to capture the sequence signal. On the other hand, single-locus methods are better suited to capture individual signals scattered across the genome (i.e., recombination has broken physical association, or they are located far enough from each other). The single-locus methods we used are likely to respond to different facets of the selective process: while raw allele frequency differences (AFD) may become apparent early in the divergence process, F-statistics-based methods rest on ratios of between populations / within-populations variances, which may increase only after the selective process has pushed each population near fixation. We can safely suggest to use more than one method when a hypothesis on selection dynamic is lacking.

Our historical demography results are mostly consistent with Lander et al. (2011), both for population split times and for the upper boundaries of population size confidence intervals. Nevertheless, our medians and modes for population sizes are an order of magnitude larger, and the lower boundaries an order of magnitude smaller, so that globally, we tend to obtain larger estimates than Lander et al. (2011). The duration of the period over which population size would have stayed at a minimum is in the order of 90–99 generations, placing it approximately within historical times. A slight difference with Lander et al. (2011) is that the lower boundary of our growth rate estimates also includes contraction. However, timing of the most ancient events (post-glacial expansion) is poorly estimated. Interestingly though, Milesi et al. (2023) detect very stable effective population sizes for beech over the last hundreds of thousands of years, at the continental level, somehow pushing expansion events far back in the past, supporting our results. While our approach is likely to carefully control divergence in allele frequencies stemming from purely neutral, demographic processes (i.e., allele surfing), we cannot rule out that the demographic parameters estimated can be biased by the presence of undetected background selection (Charlesworth et al., 1993), since strongly deleterious, low-frequency alleles could have been interpreted as signatures of expansion (Ewing and Jensen 2016; Johri et al. 2020, 2022). This happens because intermediately deleterious mutations accumulate in a non-neutral fashion in the “rare alleles” classes of an SFS in the context of non-equilibrium processes. During demographic expansions novel mutations are generated, with the risk of inflating the “rare alleles” classes of the SFS. *F sylvatica* genome size (542 Mb, Mishra et al. 2018) and mutation rate (9.5 × 10^−10^, Milesi et al. 2023) allow us to infer that each gamete carries ≈ 0.5 new mutations. We can expect ≈ 5000 novel mutation having occurred in the population of Mont Ventoux over the last 150 years given that its estimated effective population size *N*_*e*_ is the number of gametes that constitute the next generation (5000), the number of elapsed generation since the reforestation process (2), and the number of novel mutations for each gamete (0.5). In addition to this, the use of SFS-derived statistics to infer demography has been criticised because of its high sensitivity to fluctuations in SFS (Myers et al. 2008; Lapierre et al. 2017). Another limitation of our work is that we mostly focused on allele frequency shifts rather than changes in allele frequency covariances, which are known to be more involved in the early stages of adaptation (Le Corre and Kremer 2003, 2012; Cubry et al. 2022). This may explain the low number of outliers detected here.

The second (evolutionary) take-home message can be derived from the combination of the detection of divergence outliers and the estimation of population divergence times: the different stands have become separated only a few generations ago, yet signatures of the effect of divergent selection appear in the data. This suggests that population recolonisation and the occupation of novel environments was followed by selective processes strong enough to provoke population divergence. First, we showed that separate parts of the historical relict population (pairs N3-S5 and N4-S5) show signatures of adaptive molecular divergence. This suggests that geographical separation and environmental divergence can establish adaptation-driven population divergence even if the populations are quite close geographically (here, S5 suffers less summer drought stress than N3-N4, but undergoes more intense frost; Table 1). Second, newly colonised areas (stand S1) showed signatures of adaptive divergence. As S1 was generated by recolonisation over the last one-and-a-half century at most, this suggests that adaptive processes can be very rapid, likely driven by the very different environmental conditions experienced by S1 relative to the three historical relict populations. Third, and quite surprisingly, sub-populations within the same part of the historical relict zone (N3 and N4), but experiencing slightly different environmental conditions, also showed adaptive divergence, despite the conservativeness of the tests. So, stable sub-populations, undergoing environmental contrasts, putatively undergo divergence even when they are geographically contiguous.

We expected that the loci that diverge between stand par among an environmental gradient are associated with phenotypic traits that usually vary in these landscape contexts, like budburst (Gauzere et al. 2020) or response to drought (Bontemps et al. 2017). The only gene with a possible relationship to phenology is a putative LOV domain-containing protein (Ito et al. 2012; Glantz et al. 2016), belonging to a family of flavoproteins that modulate photosensitivity. We can also hypothesise that the identified isoflavone reductase homolog (Cheng et al. 2015) may be involved in this pathway. We also noticed that four genes have been studied in the context of plant defense from different kind of pathogens (endo-1,3-beta-glucosidase, Sperisen et al. 1991, and Kebede and Kebede 2021; probable disease resistance protein RF9, Milc et al. 2019; isoflavone reductase homologue, Cheng et al. 2015) and two are involved in the response to oxidative (GPDHC1; Shen et al. 2006) and osmotic stress (NAC domain-containing protein; Duval et al. 2002).

A third (evolutionary) message is that novel adaptation was fuelled by the genetic variation already present in the historical relict zone, through changes in allele frequencies between the source and the derived stands. This is an indication that divergent selection can be detected over both short *distances* (as already reported in Audigeos et al. 2013; Lind et al. 2017; Brousseau et al. 2021; Scotti et al. 2023; Budde et al. 2023) and short *times*, and that populations can diverge quickly from standing variation. The same stands were shown to also be genetically differentiated at adaptive traits (Gauzere et al. 2020), which reasonably can only have diverged from the same genetic background and over the same time span as genomic loci.

## Conclusions

The adaptive processes ongoing in the European beech population of Mont Ventoux have been the object of several studies (Lalagüe et al. 2013; Csilléry et al. 2014; Gauzere et al. 2020) relying on candidate gene and phenotype data. Here we take a genome-wide perspective (even though restricted to exons) to study adaptive processes. We integrated genomic data with a reconstruction of the forest demographic history building on the work of (Lander et al. 2011, 2021) and exploited the recently assembled genome of *Fagus sylvatica* from Mishra et al. (2022) to map our polymorphisms and retrieve functional annotations.

We were able to retrieve divergence signals, both at the single-SNP and the gene level, possibly caused by local environmental pressures, which can be detected even on very small spatial scales (~150 m) but also between newly expanded and old-growth forests. This indicates that, while overall genome-wide divergence is as small as expected for such short geographical distances, selection can target portions of the genome and cause divergence, as already shown for phenological characters. Such observations suggest that within-population genetic diversity has been a powerful reservoir of adaptive potential, which allowed expanding cohorts to adapt to new conditions over very few generations. Such genetic properties may contribute to help forests adjust to climate upheavals, and are a potential ally for establishing forest management practices favouring adaptation.

### Supplementary information

Supplementary Material

## Data Availability

Data and scripts used to analyse them are available at 10.57745/BE4HIG.
