# Supplementary material for: Rolling down that mountain: microgeographical adaptive divergence during a fast population expansion along a steep environmental gradient in European beech
Source: Heredity (Edinb). 2024 Jun 18;133(2):99–112. doi: 10.1038/s41437-024-00696-z (PMC11286953; doi:10.1038/s41437-024-00696-z)
Supplement: Supplementary file 1 — Supplementary Material [file 41437_2024_696_MOESM1_ESM.pdf]

Supplementary Methods 1

We fed BLAST 2.9.0 (Altschul *et al.*, 1990; Zhang *et al.*, 2000) To obtain the positions of target regions and SNPs on the reference genome, we proceeded as follows. We first extracted the best hit for each target region and retrieved the positions of each nucleotide in the target regions relative to the reference scaffolds (including gaps in the alignment). We then build a correspondence table providing the position of each nucleotide of each target region on reference scaffolds (target regions with no hit were removed). Using this table, target regions positions of SNPs were converted into scaffold positions.

We performed the mapping of our SNP dataset using the Bhaga reference nuclear genome of *F. sylvatica* in fasta format as a reference genome. We built a database with the following line

makeblastdb -in /home/pc/Documenti/FagusLab/Fagus-sylvatica_DE-Genome/Fs_NuclearGenome/Bhaga_genome.fasta -parse_seqids -blastdb_version 5 -title "Fagus sylvatica German Nuclear" -dbtype nucl -out Fsylvatica_DE_NuclearGenome

We then proceed to align our 11,527 target regions with BLAST 2.9.0+ using the command

blastn -db Fsylvatica_DE_NuclearGenome -query /home/pc/Documenti/FagusLab/Fagus_pc_Turin/Fs_contigsK81wrap.fa -out Fagus_0.txt -max_hsps 6 -dust no -outfmt "0 qseqid sseqid qstart qend sstart send gapopen gaps pident nident mismatch evalue length"

We used a suite of script to obtain first the alingment of our target regions against the reference genome, then map each polymorphisms from the sequencing experiment to the Bhaga's genome.
We fed the BLAST+ output in format 0 to our first script to obtain the region' sequence aligned to the reference genome. The script identifies the start of each query and
checks the orientation of the alignment between the query and the genomic subject.
The pipeline writes one file.txt for each target region with a hit. In this way we retrieved the alignment for 11,503 of the BLASTed target region.
In a second scrip we finaliised the mapping of each polymorphism. We imported in RStudio the vcf of the 35,997 filtered SNPs in 9,055 target regions by vcfR *read.vcfR()* (Knaus and Grünwald, 2017). This is indeed our starting SNP dataset.
The compilation of the map of the correspondences between queries and subjects is performed within a loop. that provides a dataframe of the mapped SNPs. At the end of the loop we retrieved 80 unmapped SNP. We properly called this mapping dataframe "rosettaStone".

In the next steps we removed the SNPs (rows) whose genomic position was the same as another one. We found that 154 SNPs had their position duplicated at least twice. These SNP-clones were harboured in 390 target regions. To avoid artefacts, we also removed all SNPs of these target regions that contain at least one SNP with redundant genomic position, thus dropping further 1108 SNPs.
The resulting dataset was of 34,889 SNPs harboured in 8,791 and mapped on the *F. sylvatica* reference genome provided by (Mishra *et al.*, 2021).

We used the information concerning the genomic region and the position of all the mapped SNP to produce a tab-delimited table. We feed this table together with the filtered vcf to VCFTOOLS 0.1.16 (Danecek *et al.*, 2011) to obtain a vcf containing only the variants which had been mapped to an unequivocal position in the genome. A bash file was used with the following line:

vcftools --vcf fagus96filtered.vcf --exclude-positions CHROMandPOS2drop.txt --recode --recode-INFO-all --out fagus96golden

Using a reference table of the individual belonging to each stand, we produced two new files containing each the individuals of each pair of stands as well a vcf file pooling the variants of all the individuals

Script for mapping each nucleotide on the scaffold

Here we build a reference table from the BLAST+ output and use it to map our whole dataset to the reference genome. BLAST+ was run with the aforementioned command line:

```
blastn -db Fsylvatica_DE_NuclearGenome -query /home/pc/Documenti/FagusLab/Fagus_pc_Turin/Fs_contigsK81wrap.fa -out Fagus_0.txt -max_hsps 6 -dust no -outfmt "0 qseqid sseqid qstart qend sstart send gapopen gaps pident nident mismatch evalue length"
blastn -db Fsylvatica_DE_NuclearGenome -query /home/pc/Documenti/FagusLab/Fagus_pc_Turin/Fs_contigsK81wrap.fa -out Fagus_0.txt -max_hsps 6 -dust no -outfmt "0 qseqid sseqid qstart qend sstart send gapopen gaps pident nident mismatch evalue length"
```

Randomness of distribution of target sequences

To check whether the sequenced genomic regions were randomly distributed across the genome, their positions were compared to the expectation obtained following a Poisson distribution. Under a Poisson process, the ratio of variance to mean is χ^2-distributed with one degree of freedom; when the ratio of variance to mean equal to 1.0 , the studied process that causes object to be scattered in space or time follows perfectly a Poisson distribution; lower values suggest that it scatters the object more randomly than a Poisson process, higher values suggests that it clump them together instead. This property can be used to test whether the observed distribution follows a Poisson process. To test whether the sequenced genomic regions were randomly distributed over the genome, we counted the number of sequenced regions in 1,000,000-bp genomic windows, computed the mean and variance of counts, computed their ratio and applied the above χ^2 test.

REFERENCES

Altschul SF, Gish W, Miller W, Myers EW, Lipman DJ (1990). Basic Local Alignment. *J Mol Biol* **215**: 403–410.

Danecek P, Auton A, Abecasis G, Albers CA, Banks E, DePristo MA, *et al.* (2011). The variant call format and VCFtools. *Bioinformatics* **27**: 2156–2158.

Knaus BJ, Grünwald NJ (2017). vcfr: a package to manipulate and visualize variant call format data in R. *Mol Ecol Resour* **17**: 44–53.

Mishra B, Ulaszewski B, Ploch S, Burczyk J, Thines M (2021). A circular chloroplast genome of fagus sylvatica reveals high conservation between two individuals from Germany and one individual from Poland and an alternate direction of the small single-copy region. *Forests* **12**: 1–7.

Zhang Z, Schwartz S, Wagner L, Miller W (2000). A greedy algorithm for aligning DNA sequences. *J Comput Biol* **7**: 203–214.

Supplementary Methods 2

Detailed methods for the identification of the demographic model

We estimated demographic parameters for the four stands by applying FastSimCoal2 v2.7.0.5 (Excoffier and Foll, 2011) to folded joint pairwise site frequency spectra (SFS) for each pair of stands, from now on called 2D-fSFS.

2D-fSFS computation

To compute the observed 2D-fSFS both at the genomic region and the scaffold level we fed the *vcfSieve()* function (Scotti et al., 2023) a VCF file containing the position of the SNPs (on the genomic regions and on the scaffolds, respectively), a fasta file of the genomic reference assembly or the scaffold sequences, and a table mapping each individual to its stand. After having counted the alternative and reference allele for each population, we imputed the missing data with the custom function *imputing(),* which takes the counts of each allele at a locus in a population and the maximum number of valid genotypes at any locus in a population to return a vector of the counts for each allele of the imputed SNPs. Next, the global minor allele at each SNP was identified with the custom function *minAlleleCounts()*, which returns a vector of (global) minor allele counts for each population. In the final step, the 2D-fSFS were produced by combining the vectors of (global) minor allele counts for each pair of populations (the numbers of invariant sites in the sequences, i.e. the total length of the target regions minus the number of SNPs, were added to the [0] and the [0,0] cells columns). The R script used to produce 2D-fSFS is the n°17 available at the DataVerse repository.

Figure SM2-1. Workflow of production of demography-based simulated 2D-fSFS

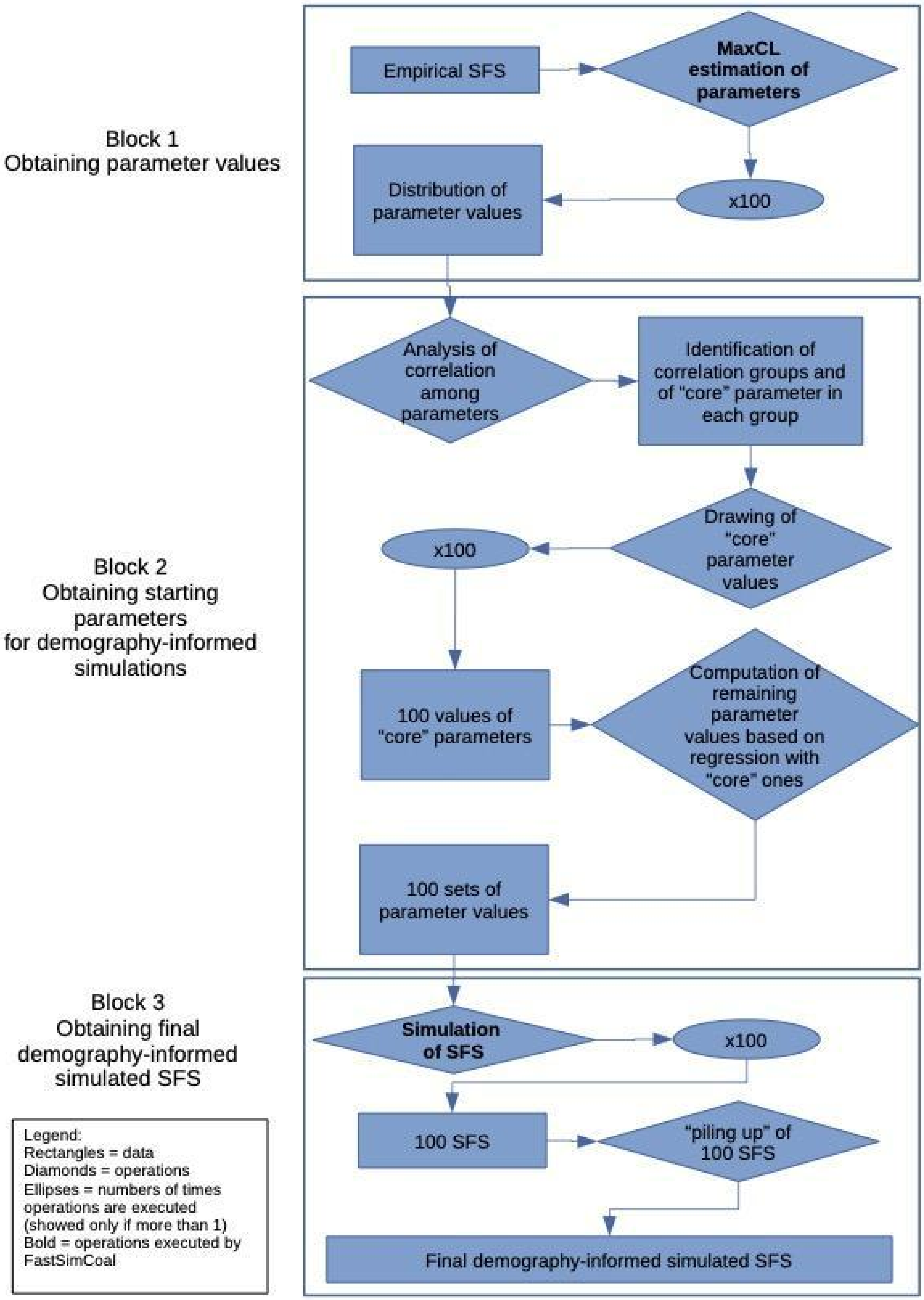

Estimation of demographic parameters and simulation of a demography-informed joint 2D-fSFS

FastSimCoal2, version 2.7.5 (Excoffier and Foll, 2011) was used twice, to (1) estimate the demographic parameters under a given demographic scenario using the observed 2D-fSFSs, and (2) generate a simulated 2D-fSFSs based on the most-likely demographic scenarios, after a treatment of the output of (1) that is described below (see Figure 1 for the workflow). Box 1 reports the master (TPL) file coding for the demographic events in FastSimCoal (see also Figure 2 in the main document for a graphical description of the events).

Box 1: FastSimCoal master file (TPL) code describing the demographic model

```
//Number of population samples (demes)
4 samples to simulate
//Population effective sizes (number of genes)
POPSIZE0
POPSIZE1
POPSIZE2
POPSIZE3
//Sample sizes
40 0 -0.05253664
50 0 -0.04238771
50 0 -0.04314575
52 0 -0.04139063
//Growth rates : negative growth implies population expansion
GROWTHRATE0
GROWTHRATE0
GROWTHRATE0
GROWTHRATE0
//Number of migration matrices : 0 implies no migration between
demes 1
//migration matrix
0.000 M01 M02 M03
M12 0.000 M12 M13
M02 M12 0.000 M23
M03 M13 M23 0.000
//historical event: time, source, sink, migrants, new size, growth rate,
migr. matrix NOTE: order of event 1 and 2 to be
estimated 8 historical event
TIME1 0 1 1 1 GROWTHRATE0 0
TIME1 0 0 0 0 GROWTHRATE0 0
TIME2 2 1 1 1 GROWTHRATE0 0
TIME2 2 2 0 0 GROWTHRATE0 0
TIME3 3 1 1 1 GROWTHRATE0 0
TIME3 3 3 0 0 GROWTHRATE0 0
TIMEA 1 1 1 1 0 0 nomig
TIMEB 1 1 0 1 GROWTHRATE1 0
TIMEC110100
TIMED 1 1 0 1 GROWTHRATE2 0
TIMEE110100
//Number of independent loci
[chromosome] 1 0
//Per chromosome: Number of linkage
blocks 1
//per Block: data type, num loci, rec. rate and mut rate + optional
parameters
```

```
FREQ 1 0 1e-8 OUTEXP
```

Estimation of demographic model parameters

We used the six 2D-fSFSs as input to the maximum-likelihood parameter estimator of Fastsimcoal2 v2.7.5 (with the following parameters: number of independent loci 1, chromosome 0, number of linkage blocks per chromosome 1, data type FREQ, num loci 1, recombination rate 0, mut rate 10-8; -n 100000 -M -c 4). We repeated the estimation step 100 times to obtain a distribution of values for each parameter, to account for uncertainties in parameter estimation (Figure 1, Block 1). See Table 1 for the 100 maximum likelihood sets of parameters and Figure SM2-2 for their distribution.

Analysis of the covariation structure of parameter values

Prior to the sampling of sets of parameter values for the simulation of demography-informed 2D-fSFS, we examined (a) the similarity of parameter sets obtained with the 100 estimates and (b) the covariation among parameters (Figure 1, Block 2).

To check whether the hundred parameter sets belonged to the same group (i.e., the most likely scenarios from all estimations were a random draw from a single “type” of scenario) or formed multiple groups (i.e., more than one “type” of scenario was obtained), we applied a principal component analysis, in which each estimation was an object, and each parameter a variable describing the objects, with the function *dudi.pca()* of Adegenet 2.1.10 (Jombart, 2008). We retained the two first principal components, explaining 27.69 and 14.35 % of the variance, respectively. According to these axes, we identified two clusters of parameter sets, containing 64 and 30 parameter sets each (six parameter sets did not fall in a cluster; Figure 2).

Figure SM2-2. Distribution of estimated parameter values and principal component analysis of estimated parameter sets

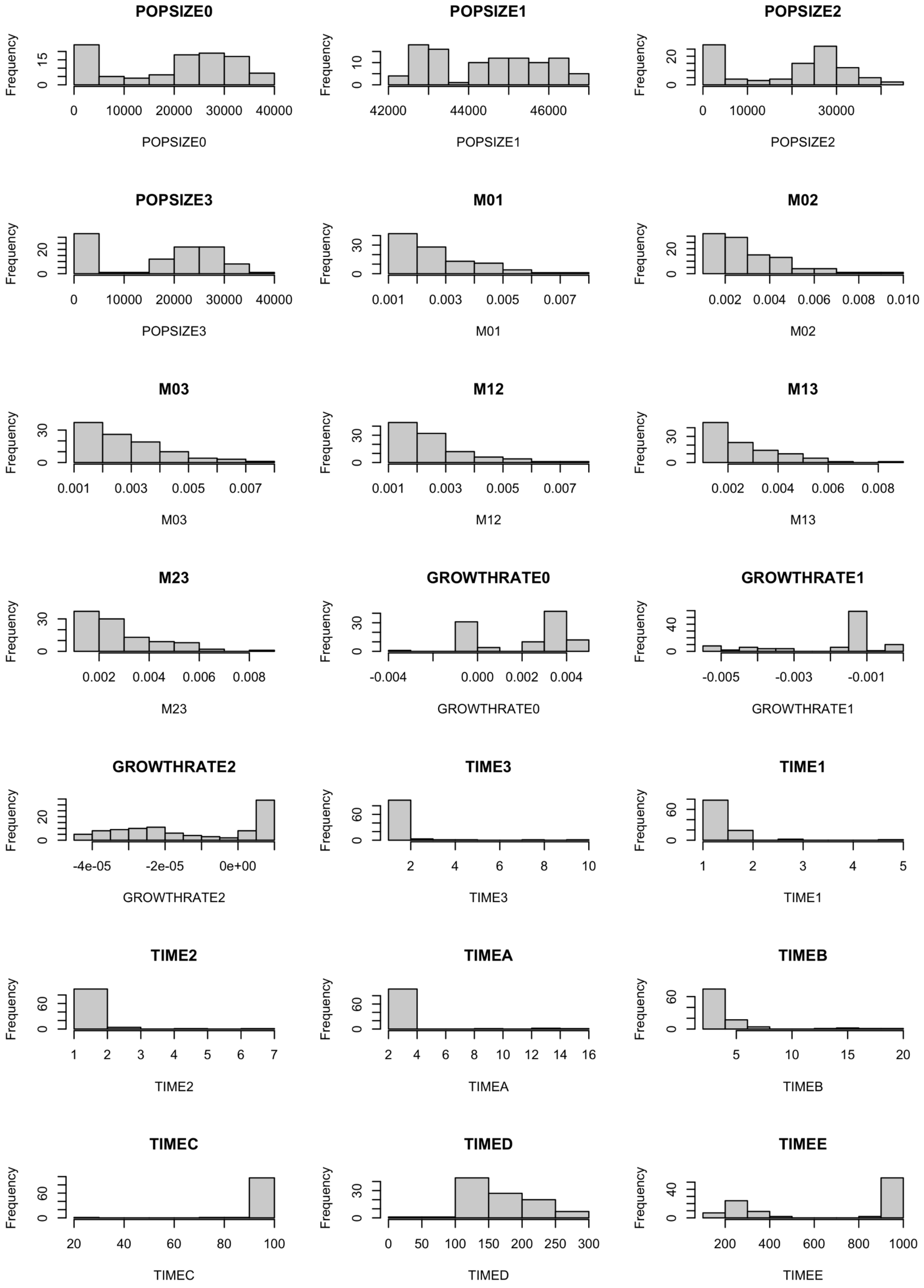

Figure SM2-2 (continued)

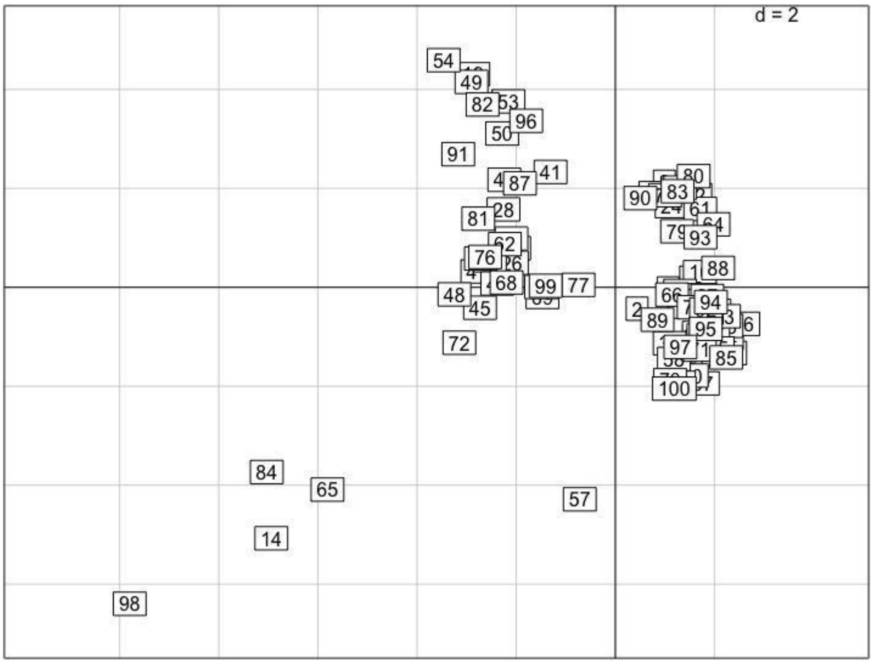

This indicated that the maximum likelihood estimator identified two peaks of likelihood, corresponding to the two groups of parameter sets.

We then proceeded to inspect the covariation among parameter values within each cluster of parameter sets, for all pairs of parameters (21*20 / 2 = 210 pairs of parameters) using the script n°11. We inspected the P-value of the slope of the correlation (after Bonferroni correction for multiple tests) and identified, in each of the two clusters of parameter sets identified by PCA, the groups of correlated parameters shown in Figure 3.

Figure SM2-3. Groups of correlated parameters in each of the two clusters of parameter sets

```
                                        TIME2 - TIME3 - TIMEC
                                                  \         /
                                   GROWTHRATE1 - GROWTHRATE0 -
Cluster1 Group 1:                  M23
                                       |                 |
                                     TIMEB           POPSIZE0
```

```
Cluster 1 Group 2:   GROWTHRATE2 -- TIMED
                              \/
                             TIMEE
```

```
Cluster 2 Group 1: GROWTHRATE1 - TIMEB
```

```
Cluster 2 Group 2: TIMEE - GROWTHRATE2 - TIMED
```

In each group of correlated parameters we identified a "core" parameter, based on the strength of correlation with all others; the "core" parameters are underlined in Figure 3.

Calculation of priors for the final simulations

In the next step, we used FastSimCoal again one hundred times, to produce one hundred joint 2D-fSFS. We drew starting sets of parameters for each simulation from the above distributions of parameter values; however, because (a) parameter sets were grouped in two clusters, and (b) at least some of the parameters were correlated within each cluster, we could not draw each parameter independently from the others, which would have lead to implausible combinations of parameter values. To obtain input sets of parameter values that took into account parameter covariation structure, we proceeded as follows (Figure 1, Block 2):

(a) In each cluster of parameter sets, we draw values for all uncorrelated parameters and for the "core" parameter in each group of correlated parameters from a uniform

distribution, having as boundaries the minimum and maximum of the distribution of each parameter (Table 1)

Table 1. Sets of parameter estimates

Note that the signs of the GROWTHRATE parameters have been flipped to make them more understandable. They must be filpped again to write the TPL input file of FastSimCoal because the software package works in a coalescent framework (*ie*. backward in time) : hence it requires changes in population size to be written with the opposite sign. A FastSimCoal input file has demographic expansiion written with a negative sign as if they were contraction, and demographic contraction written with a positive sign as if they were expansions.

**estimation run**	**POPSIZE0**	**POPSIZE1**	**POPSIZE2**	**POPSIZE3**	**M01**	**M02**	**M03**	**M12**	**M13**	**M23**
**1**	16989	45528	30362	29609	0.0020583	0.0043380	0.0027911	0.0015440	0.0034927	0.0062298
**2**	26047	43316	27738	20339	0.0025403	0.0046100	0.0023790	0.0050612	0.0037185	0.0047248
**3**	26472	45229	35600	25555	0.0013623	0.0022712	0.0025265	0.0041012	0.0022865	0.0041127
**4**	10149	44181	3815	2506	0.0019135	0.0044756	0.0037832	0.0018199	0.0046790	0.0023165
**5**	38529	42804	23590	20928	0.0030556	0.0032360	0.0016559	0.0017082	0.0027904	0.0044508
**6**	32562	44733	36469	17790	0.0039060	0.0042928	0.0021892	0.0031359	0.0017087	0.0023789
**7**	31552	42813	34793	27065	0.0017431	0.0015963	0.0022664	0.0019327	0.0019696	0.0017608
**8**	18521	45385	18179	24056	0.0012447	0.0040617	0.0018996	0.0041371	0.0019570	0.0016710
**9**	32290	42238	31404	24685	0.0022514	0.0027231	0.0024361	0.0017591	0.0021267	0.0012114
**10**	24854	45068	29711	18825	0.0026589	0.0022974	0.0010994	0.0027069	0.0038118	0.0019298
**11**	30450	42147	25998	27523	0.0028038	0.0014824	0.0013351	0.0019766	0.0020182	0.0014671
**12**	26056	46156	28988	20145	0.0026577	0.0017353	0.0049492	0.0021254	0.0019092	0.0023340
**13**	25902	45942	30498	19562	0.0017231	0.0023839	0.0020568	0.0022376	0.0049702	0.0016601
**14**	13539	43008	7669	6088	0.0073210	0.0021879	0.0017458	0.0047151	0.0014368	0.0024586
**15**	31412	43410	25370	24792	0.0012894	0.0016184	0.0019888	0.0021464	0.0013711	0.0033855
**16**	25431	46508	12921	31442	0.0017029	0.0017717	0.0043035	0.0015584	0.0018314	0.0018543
**17**	25842	42496	30582	36050	0.0046875	0.0031449	0.0019500	0.0027870	0.0019655	0.0023718
**18**	27378	43294	24386	25837	0.0021043	0.0036794	0.0022973	0.0018362	0.0019832	0.0014659
**19**	593	46171	1474	1497	0.0035980	0.0052004	0.0025355	0.0033245	0.0082860	0.0020434
**20**	29151	44192	29959	18003	0.0017957	0.0026537	0.0030256	0.0013378	0.0013453	0.0016164

**21**	3258	45495	11498	1381	0.0034407	0.0038675	0.0037998	0.0017596	0.0014105	0.0057621
**22**	25134	45751	28360	28003	0.0018567	0.0049049	0.0032898	0.0029902	0.0022689	0.0020522

**23**	22319	42913	26485	20117	0.0014934	0.0023277	0.0015871	0.0011440	0.0019387	0.0029899
**24**	23459	43043	27262	16387	0.0040465	0.0017998	0.0020397	0.0016468	0.0042410	0.0041017
**25**	566	44985	1590	388	0.0040010	0.0032986	0.0046089	0.0013286	0.0027434	0.0019385
**26**	2954	43510	6725	3173	0.0017468	0.0028167	0.0032586	0.0027336	0.0023762	0.0080734
**27**	26717	46605	25530	26239	0.0018510	0.0020506	0.0015025	0.0017409	0.0016274	0.0015850
**28**	2140	45640	623	1004	0.0022213	0.0015114	0.0058438	0.0031403	0.0056911	0.0025388
**29**	39360	43110	25235	32272	0.0014261	0.0052391	0.0049951	0.0014408	0.0014744	0.0017979
**30**	22196	46007	20447	21326	0.0035082	0.0018678	0.0019596	0.0017360	0.0012007	0.0036085
**31**	14161	44235	1806	1003	0.0036467	0.0042919	0.0052109	0.0028586	0.0026894	0.0029025
**32**	23221	45373	20627	28923	0.0027837	0.0022065	0.0046309	0.0016914	0.0027020	0.0053857
**33**	26989	42789	28252	28357	0.0027171	0.0017489	0.0043470	0.0016671	0.0028915	0.0026331
**34**	32982	42572	33184	21957	0.0026190	0.0015667	0.0028711	0.0014619	0.0023223	0.0011679
**35**	37167	43295	28148	24905	0.0038220	0.0028957	0.0031191	0.0024254	0.0013588	0.0015876
**36**	39212	42990	44591	34366	0.0016799	0.0025218	0.0012917	0.0026289	0.0057725	0.0014469
**37**	33126	43224	34425	33428	0.0021276	0.0022931	0.0016829	0.0030512	0.0019360	0.0014682
**38**	34807	42760	22782	20131	0.0031141	0.0019012	0.0036954	0.0019635	0.0024517	0.0013830
**39**	24816	44918	25742	26826	0.0019257	0.0019048	0.0040194	0.0018155	0.0021206	0.0059516
**40**	29137	46083	41360	29487	0.0012344	0.0019339	0.0037077	0.0036903	0.0013595	0.0037399
**41**	12091	44547	3330	198	0.0020814	0.0048290	0.0032809	0.0023963	0.0015015	0.0013239
**42**	2200	43269	2747	891	0.0018455	0.0035443	0.0051614	0.0014193	0.0014946	0.0017384
**43**	729	44909	1958	685	0.0016936	0.0061993	0.0024310	0.0036428	0.0015391	0.0024626
**44**	30586	43050	39629	19666	0.0030414	0.0029528	0.0032341	0.0014917	0.0019562	0.0013256
**45**	7463	44445	5870	1612	0.0043374	0.0018261	0.0022276	0.0018038	0.0024868	0.0046656
**46**	32039	44509	32863	22319	0.0025176	0.0021544	0.0013058	0.0016296	0.0015857	0.0011813
**47**	26545	42686	21085	26076	0.0012089	0.0029975	0.0014775	0.0015137	0.0015625	0.0019558
**48**	1757	42543	2387	1152	0.0040532	0.0031800	0.0023983	0.0055458	0.0024429	0.0026792

**49**	1148	46009	685	599	0.0052405	0.0048163	0.0071654	0.0032883	0.0040012	0.0038882
**50**	8604	46106	1363	281	0.0046000	0.0036715	0.0016786	0.0022834	0.0013144	0.0043421
**51**	2383	45650	1048	1411	0.0045647	0.0015937	0.0017985	0.0027232	0.0043129	0.0019610
**52**	606	44330	2772	1107	0.0017610	0.0062713	0.0019017	0.0044884	0.0039619	0.0023022
**53**	637	45329	26202	993	0.0057031	0.0070853	0.0037001	0.0025474	0.0045334	0.0026198
**54**	1445	45718	1497	998	0.0022732	0.0081198	0.0066850	0.0066890	0.0058613	0.0058043
**55**	24437	42790	25497	26288	0.0015893	0.0017789	0.0013608	0.0020315	0.0027407	0.0016435
**56**	22860	45134	32229	25096	0.0029346	0.0014134	0.0027688	0.0023446	0.0016268	0.0017622
**57**	24250	45804	25480	24720	0.0045381	0.0020145	0.0041490	0.0048301	0.0025608	0.0015551
**58**	18521	44316	22168	26212	0.0016522	0.0021541	0.0014966	0.0013745	0.0023398	0.0023172
**59**	21073	44917	26805	25620	0.0012987	0.0016898	0.0067630	0.0013219	0.0015491	0.0034061
**60**	27614	46345	16646	25474	0.0012758	0.0014478	0.0030697	0.0015337	0.0012328	0.0035035
**61**	28319	46435	22170	21277	0.0022060	0.0014689	0.0023313	0.0014445	0.0038074	0.0021562
**62**	596	42973	2551	1744	0.0039696	0.0021611	0.0016827	0.0070776	0.0046158	0.0021087
**63**	23958	46420	26347	27309	0.0025729	0.0029598	0.0033444	0.0024139	0.0038419	0.0036227
**64**	31818	46026	30581	16811	0.0021585	0.0016629	0.0019614	0.0022000	0.0018509	0.0021604
**65**	1474	42836	3474	4176	0.0056961	0.0042862	0.0016088	0.0022273	0.0018431	0.0028587
**66**	22807	44271	29763	26800	0.0014303	0.0058171	0.0023528	0.0030410	0.0044489	0.0023523
**67**	31558	46345	25754	23881	0.0016230	0.0042054	0.0032406	0.0016253	0.0013446	0.0022458
**68**	3104	42525	4533	689	0.0053513	0.0063969	0.0016686	0.0024411	0.0029649	0.0013670
**69**	3430	42565	1305	1549	0.0016139	0.0026014	0.0032062	0.0028643	0.0017127	0.0016380
**70**	31866	42976	23867	28510	0.0021052	0.0036581	0.0030075	0.0028746	0.0012147	0.0035692
**71**	23306	43381	26951	33062	0.0015904	0.0034049	0.0012560	0.0012661	0.0014192	0.0040118
**72**	1201	42967	1578	915	0.0017990	0.0037523	0.0025709	0.0020915	0.0051796	0.0051864
**73**	25395	45277	30850	28667	0.0018222	0.0021068	0.0025303	0.0015593	0.0013862	0.0016543
**74**	30081	44873	28019	19349	0.0014997	0.0019268	0.0021025	0.0018074	0.0033126	0.0022055

**75**	21646	45023	16657	22481	0.0013354	0.0021039	0.0021000	0.0026977	0.0017787	0.0031661
**76**	2296	43021	4743	1008	0.0025255	0.0019039	0.0055556	0.0055895	0.0043193	0.0018952
**77**	19737	43024	3378	395	0.0016512	0.0029661	0.0017804	0.0021584	0.0028365	0.0037980
**78**	37129	46683	35952	19962	0.0042561	0.0017891	0.0016475	0.0029156	0.0035167	0.0015859
**79**	17957	44791	20016	25031	0.0022729	0.0047009	0.0019303	0.0023754	0.0017047	0.0030131
**80**	21155	45635	24314	24072	0.0017629	0.0015213	0.0027081	0.0018352	0.0019748	0.0031784
**81**	2007	45892	5388	4048	0.0022385	0.0028668	0.0032082	0.0052066	0.0036893	0.0054747
**82**	3502	46537	990	508	0.0036601	0.0035956	0.0069427	0.0049410	0.0016463	0.0020526
**83**	23603	45733	36248	31010	0.0015793	0.0055258	0.0013522	0.0027340	0.0042336	0.0022363
**84**	2936	44110	3245	4175	0.0061206	0.0069608	0.0022703	0.0021071	0.0030737	0.0066139
**85**	36287	42470	20572	33137	0.0022954	0.0025316	0.0017723	0.0014137	0.0024570	0.0013042
**86**	9537	44500	4127	849	0.0016202	0.0041653	0.0012225	0.0028367	0.0015374	0.0025766
**87**	9118	45300	1011	289	0.0046865	0.0025436	0.0018604	0.0011484	0.0057118	0.0014612
**88**	35492	46615	26137	23817	0.0031421	0.0013946	0.0037557	0.0014271	0.0013522	0.0016244
**89**	31521	46237	29250	17017	0.0028240	0.0014380	0.0015756	0.0027498	0.0030119	0.0021988
**90**	26516	45423	16779	30739	0.0019732	0.0039980	0.0018366	0.0031421	0.0023173	0.0040916
**91**	2618	42979	1142	1190	0.0032097	0.0035758	0.0047944	0.0016340	0.0068392	0.0051327
**92**	27476	45254	28169	24592	0.0020673	0.0016496	0.0018377	0.0035742	0.0015697	0.0011770
**93**	32788	44997	24218	17089	0.0018814	0.0036013	0.0021653	0.0012971	0.0015772	0.0026887
**94**	23186	44362	27867	23613	0.0013940	0.0013085	0.0026063	0.0017335	0.0033169	0.0021078
**95**	31251	43060	20058	20419	0.0015802	0.0016817	0.0022274	0.0020346	0.0020493	0.0033500
**96**	6182	44875	549	820	0.0046881	0.0015943	0.0013090	0.0034801	0.0019743	0.0049738
**97**	25394	43449	22933	24784	0.0015235	0.0029232	0.0017028	0.0016215	0.0030197	0.0020253
**98**	18483	44929	10633	10736	0.0025470	0.0095765	0.0019124	0.0018677	0.0032266	0.0053061
**99**	4837	42639	538	320	0.0029048	0.0016836	0.0047634	0.0034676	0.0016075	0.0014970
**100**	23088	43055	32477	16648	0.0014698	0.0026753	0.0035328	0.0018818	0.0016737	0.0017027

Table 1 (continued)

estimation run	TIMEA	GROWTHRATE0	GROWTHRATE1	GROWTHRATE2	TIME3	TIME1	TIME2	TIMEB	TIMEC	TIMED	TIMEE
**1**	2	3.65939e-03	-0.0012445	-3.78491e-05	1	1	1	4	99	118	982
**2**	2	3.64945e-03	-0.0012931	-2.29197e-05	1	1	1	6	98	160	984
**3**	2	-3.16348e-03	-0.0012767	-2.49656e-05	1	1	1	5	98	152	965
**4**	4	-7.44892e-05	-0.0012477	7.31034e-06	3	3	1	4	99	254	263
**5**	2	3.65292e-03	-0.0012444	-3.02971e-05	1	1	1	3	98	139	990
**6**	2	3.59345e-03	-0.0018592	5.42227e-06	1	1	1	5	99	220	258
**7**	2	2.72577e-03	-0.0012540	-3.76152e-05	1	1	1	3	98	103	987
**8**	2	3.82657e-03	-0.0049083	-2.07492e-05	2	1	1	2	99	179	953
**9**	2	3.17834e-03	-0.0012373	5.79805e-06	1	1	1	3	99	231	247
**10**	2	3.21896e-03	-0.0012516	-2.57811e-05	1	1	1	4	99	107	987
**11**	2	3.10551e-03	-0.0012426	2.41586e-06	1	1	1	4	99	230	286
**12**	2	3.42136e-03	-0.0041545	-3.11129e-05	1	1	1	2	98	113	986
**13**	2	3.54165e-03	-0.0015598	-3.77638e-05	1	1	1	4	98	126	941
**14**	15	-8.57739e-05	-9.85679e-05	7.00410e-06	8	3	2	15	99	213	228
**15**	2	2.80231e-03	-0.0012687	6.99811e-06	1	1	1	6	99	198	217
**16**	2	3.94423e-03	-0.0012540	8.48096e-06	2	1	1	3	98	284	304
**17**	2	3.74958e-03	-0.0012481	-2.62826e-05	2	1	1	4	99	103	950
**18**	2	3.18017e-03	-0.0012670	7.38622e-06	1	1	1	4	98	178	212
**19**	2	-1.07092e-05	-0.0054552	-8.10055e-06	2	1	2	3	98	213	961
**20**	2	3.04996e-03	-0.0012437	5.76172e-06	1	1	1	4	99	167	197
**21**	2	-9.47528e-05	-0.0012530	-2.07059e-05	2	2	1	3	98	131	988
**22**	2	3.11611e-03	-0.0012529	-3.50836e-05	1	1	1	4	99	115	979
**23**	2	3.29225e-03	-0.0012542	-7.32912e-06	1	1	1	4	99	130	934
**24**	2	4.43406e-03	-0.0041520	6.25413e-06	1	1	1	2	98	265	347

**25**	3	-9.73710e-05	-0.0012697	-1.20963e-05	1	1	2	6	99	205	933
**26**	3	-3.99872e-05	-0.0012541	-2.20135e-05	1	2	1	4	99	134	988
**27**	2	3.29717e-03	-0.0012742	6.30640e-06	1	1	1	5	98	171	183
**28**	2	-6.00268e-05	-0.0012677	-3.97041e-05	2	2	1	4	98	109	983
**29**	2	3.01424e-03	-0.0012771	5.24889e-06	1	1	1	5	98	251	399
**30**	2	3.35043e-03	-0.0012425	-1.22622e-05	1	1	1	3	98	168	949
**31**	2	4.38790e-05	-0.0012570	3.93832e-06	2	2	2	4	98	200	280
**32**	2	4.56250e-03	-0.0049212	-1.89041e-05	2	1	2	2	98	172	900
**33**	2	3.47192e-03	-0.0012527	-4.18159e-05	2	1	2	3	98	102	989
**34**	2	2.86011e-03	-0.0012245	2.37430e-06	1	1	1	2	99	175	231
**35**	2	2.80909e-03	-0.0012522	-2.28978e-05	1	1	1	3	98	149	990
**36**	2	2.56444e-03	-0.0012389	7.52293e-06	1	1	1	3	99	280	294
**37**	2	3.17800e-03	-0.0012560	6.62752e-06	2	1	2	5	99	146	205
**38**	2	3.13837e-03	-0.0012737	-4.07240e-05	1	1	1	6	99	110	984
**39**	2	4.18838e-03	-9.73525e-05	-1.94408e-05	2	1	2	3	93	122	983
**40**	3	3.68259e-03	-9.81820e-05	-4.06138e-05	3	1	1	6	88	108	987
**41**	2	-1.12046e-05	-0.0041465	-3.39451e-05	1	2	1	2	98	115	940
**42**	3	-7.51230e-05	-0.0012546	6.56147e-06	2	2	3	3	98	260	297
**43**	3	-9.56639e-05	-0.0038980	-2.98146e-05	2	1	2	3	99	116	983
**44**	2	3.16779e-03	-0.0012356	-2.15276e-05	1	1	1	3	99	110	989
**45**	4	1.17577e-05	-0.0012531	-2.57453e-05	3	2	2	4	99	132	978
**46**	2	2.89044e-03	-0.0012399	2.93798e-06	1	1	1	3	99	212	355
**47**	2	3.38537e-03	-0.0012527	5.69335e-06	1	1	1	4	99	271	301
**48**	4	-6.70017e-05	-0.0012658	-1.47586e-05	1	2	3	4	98	195	925
**49**	2	-5.66296e-05	-0.0043923	3.39183e-06	2	1	1	3	99	227	256
**50**	2	7.70036e-05	-0.0051841	4.13178e-06	1	2	2	2	99	233	331

**51**	3	-9.89125e-05	-0.0015673	-2.64790e-05	2	2	2	5	99	136	968
**52**	2	-9.90759e-05	-0.0012568	5.68606e-06	2	1	1	4	98	126	140
**53**	2	-2.63244e-05	-0.0054252	-2.30446e-05	2	1	1	2	99	194	982
**54**	2	-4.49863e-05	-0.0041595	-3.50477e-05	2	1	1	2	99	145	961
**55**	2	3.65994e-03	-0.0012658	-3.04842e-05	1	1	1	4	98	113	968
**56**	2	4.08564e-03	-9.90269e-05	-3.66653e-05	2	1	2	7	97	101	967
**57**	2	4.39560e-03	-6.07260e-04	-3.11187e-05	2	1	2	20	20	29	989
**58**	2	4.03106e-03	-0.0012890	-3.37076e-05	2	1	2	6	98	107	987
**59**	2	3.49009e-03	-0.0012652	-4.42021e-05	1	1	1	4	98	101	987
**60**	2	3.59124e-03	-0.0012700	-1.59022e-05	1	1	1	6	99	189	967
**61**	2	3.36868e-03	-0.0030435	9.13907e-06	1	1	1	2	98	230	251
**62**	2	-8.45663e-05	-0.0012424	-2.45494e-05	2	1	1	4	99	140	982
**63**	2	3.68435e-03	-0.0036048	5.16617e-06	1	1	1	2	98	203	314
**64**	2	3.34283e-03	-0.0033018	7.88147e-06	1	1	1	2	99	215	244
**65**	10	-1.61132e-05	-9.70363e-05	-3.31491e-05	4	2	5	13	97	103	969
**66**	2	3.08451e-03	-0.0012655	-2.30508e-05	1	1	1	3	97	111	985
**67**	2	3.27640e-03	-0.0012481	-2.85616e-05	1	1	1	3	98	115	985
**68**	3	-9.60236e-05	-0.0012494	-3.38938e-05	2	1	1	3	98	110	987
**69**	2	-8.32436e-05	-0.0012716	6.20338e-06	2	1	2	5	98	161	173
**70**	2	3.51253e-03	-9.81292e-05	-2.70080e-05	2	1	2	4	96	141	951
**71**	2	2.93198e-03	-0.0012908	8.09400e-06	1	1	1	6	98	214	267
**72**	3	-2.18556e-05	-9.83787e-05	-2.98662e-05	2	2	2	8	80	82	986
**73**	2	2.84997e-03	-0.0012386	7.03788e-06	1	1	1	3	99	151	179
**74**	2	3.31591e-03	-0.0012660	7.56279e-06	1	1	1	4	98	141	250
**75**	2	4.57024e-03	-0.0054206	6.90470e-06	2	1	2	3	99	235	491
**76**	3	-5.47277e-05	-0.0012773	-7.68344e-06	2	2	1	6	99	161	917

**77**	3	-9.28065e-05	-0.0012662	4.07823e-06	1	1	1	4	98	158	229
**78**	3	3.14029e-03	-0.0012569	-1.83695e-05	1	1	1	4	98	128	957
**79**	2	4.27334e-03	-0.0030380	-1.21278e-05	1	1	1	2	98	198	945
**80**	2	4.11070e-03	-0.0054365	6.77721e-06	2	1	1	2	99	232	429
**81**	3	-4.28258e-05	-0.0019002	-4.86390e-06	2	1	3	4	98	154	902
**82**	2	-3.11064e-05	-0.0044295	-1.49596e-06	1	2	2	2	99	179	838
**83**	2	4.42540e-03	-0.0051528	3.62001e-06	2	1	2	2	100	240	293
**84**	13	-9.07281e-05	-9.88701e-05	7.47675e-06	5	2	3	16	99	129	154
**85**	2	2.74011e-03	-0.0012541	-4.02809e-05	1	1	1	3	98	105	969
**86**	3	-6.40841e-05	-0.0012533	8.62290e-06	2	1	2	4	99	164	191
**87**	3	8.66488e-05	-0.0032914	-3.83476e-05	1	2	1	3	99	108	981
**88**	2	3.18368e-03	-0.0018482	7.84760e-06	1	1	1	3	99	221	240
**89**	2	3.81315e-03	-0.0015588	-2.96237e-05	1	2	2	5	99	116	989
**90**	2	4.13133e-03	-0.0054453	6.10273e-06	2	2	1	3	98	183	387
**91**	2	-2.28623e-05	-0.0038929	5.64256e-06	2	2	2	3	98	212	312
**92**	2	2.63217e-03	-0.0012505	-1.89469e-05	1	1	1	4	99	167	981
**93**	2	3.64495e-03	-0.0038923	-2.77599e-05	1	1	1	3	99	114	986
**94**	2	3.26369e-03	-0.0012534	8.67224e-06	1	1	1	3	98	191	225
**95**	2	3.49741e-03	-0.0012543	-2.48169e-05	1	1	1	3	98	139	986
**96**	2	-4.32325e-05	-0.0054813	8.12238e-06	2	1	1	2	98	230	275
**97**	2	3.93881e-03	-0.0012511	-1.57032e-05	2	1	2	4	99	171	926
**98**	13	-6.10154e-05	-9.88729e-05	5.21904e-06	10	5	7	17	97	194	226
**99**	3	-3.15225e-05	-0.0012855	-3.11207e-05	1	1	1	7	99	120	983
**100**	2	4.38553e-03	-9.67469e-05	8.82208e-06	2	1	2	7	93	214	226

(b) For all other parameters, values were obtained using the linear model describing their correlation with the "core" parameters:

$$B = \alpha A_1 + \beta + N(0,\sigma),$$

where α is the slope of the model, A_1 the "core" parameter, β is the intercept of the model; $N(0, \sigma)$ is the error term, drawn from a normal distribution $\sim N(0, \sigma)$, where σ is the model's residual standard deviation. Each value that was drawn was checked for consistency with the structure of the demographic model (in particular for the ranking of event times: TIMEA < TIMEB < TIMEC etc.), as well as for staying within the boundaries of estimated parameter values. Draws violating such constraints were discarded, and a new value was drawn, until reaching the required numbers of parameter sets (see script n°12).

Respectively, 68 and 32 parameter sets were drawn from the distribution and the covariation structure of each cluster of parameter sets, to match the size of each cluster (respectively made of 64 and 30 parameter sets). This way, we accounted for the relative weight of each peak of maximum likelihood, for the correlation structure of parameters, and for error in parameter estimation.

The one hundred parameter sets thus computed were used in the final phase.

Computing the final simulated joint 2D-fSFS

To compute the final joint 2D-fSFS from the newly produced one hundred parameter sets (Figure 1, Block 3), we first generated 100 PAR files from a TPL written to simulate the whole beech genome (Box 2) and the parameter sets (Table 1). We then used FastSimCoal anew, with the following arguments: *-i input* (n° of the PAR file)*.par -n1 -G -g*. By doing so we obtained SNP tables for six populations and 75,000 short sequences of length 1000 bp ("simulated target region data"), mimicking our empirical target regions. The GEN output files produced by FastSimCoal were then converted to VCF format using the script n°19 and then merged into a single VCF, which was treated exactly as the empirical VCF for all subsequent analyses..

Box 2: FastSimCoal master file (TPL) code to produce the PAR files

```
//Number of population samples (demes)
4 samples to simulate
//Population effective sizes (number of genes)
POPSIZE0
POPSIZE1
POPSIZE2
POPSIZE3
//Sample sizes
40 0 -0.05253664
50 0 -0.04238771
50 0 -0.04314575
52 0 -0.04139063
//Growth rates : negative growth implies population expansion
GROWTHRATE0
GROWTHRATE0
GROWTHRATE0
GROWTHRATE0
//Number of migration matrices : 0 implies no migration between demes
```

```
1
//migration matrix
0.000 M01 M02 M03
M12 0.000 M12 M13
M02 M12 0.000 M23
M03 M13 M23 0.000
//historical event: time, source, sink, migrants, new size, growth rate,
migr. matrix NOTE: order of event 1 and 2 to be
estimated 8 historical event
TIME1 0 1 1 1 GROWTHRATE0 0
TIME1 0 0 0 0 GROWTHRATE0 0
TIME2 2 1 1 1 GROWTHRATE0 0
TIME2 2 2 0 0 GROWTHRATE0 0
TIME3 3 1 1 1 GROWTHRATE0 0
TIME3 3 3 0 0 GROWTHRATE0 0
TIMEA 1 1 1 1 0 0 nomig
TIMEB 1 1 0 1 GROWTHRATE1 0
TIMEC110100
TIMED 1 1 0 1 GROWTHRATE2 0
TIMEE110100
//Number of independent loci
[chromosome] 750 0
//Per chromosome: Number of linkage
blocks 1
//per Block: data type, num loci, rec. rate and mut rate + optional
parameters
DNA 1000 9.5e-10 1e-8 OUTEXP
```

References

Excoffier, L., Foll, M., 2011. fastsimcoal: a continuous-time coalescent simulator of genomic diversity under arbitrarily complex evolutionary scenarios. Bioinformatics 27, 1332–1334. https://doi.org/10.1093/bioinformatics/btr124

Jombart, T., 2008. Adegenet: A R package for the multivariate analysis of genetic markers. Bioinformatics 24, 1403–1405. https://doi.org/10.1093/bioinformatics/btn129

Scotti, I., Lalagüe, H., Oddou-Muratorio, S., Scotti-Saintagne, C., Ruiz Daniels, R., Grivet, D., Lefevre, F., Cubry, P., Fady, B., González-Martínez, S.C., Roig, A., Lesur-Kupin, I., Bagnoli, F., Guerin, V., Plomion, C., Rozenberg, P., Vendramin, G.G., 2023. Common microgeographical selection patterns revealed in four European conifers. Molecular Ecology 32, 393–411. https://doi.org/10.1111/mec.16750

Supplementary Methods 3

Detailed methods for outlier search

Subsetting of data for outlier search tests

Bamova: The script n°20 from vcfSieve was fed with these vcfSieve objects to produce an allele-count data format which will be then used for the analyses with Bamova (Gompert and Buerkle, 2011).

Detailed methods for G2D tests

We proceeded into generating the expected 2D-SFSs both at the target region and the scaffold levels. We resampled the observed allele counts using the script n°15 to get only one allele count for each locus , then the counts were grouped either by target region and window within scaffolds. AFter having parsed the scaffolds, we built the chromosomal windows so that each one contains approximately 100 SNPs. Considering that the genome length of *F. sylvatica* is 500 M, each window will be (100 * 500 M) / 40,000 bp = 1,323,157 bp. The analysis of the window-level 2D-SFS will allow us to unravel features concerning the genomic metastructuring of our panel of SNPs and provide us hints on the genetic architecture of exonic sequences. We produced the two sets of expected 2D-SFS in a similar fashion as the observed ones by looping over the joint vectors of target region overall allele counts for each pair of populations and adding the monomorphic loci for the [0] and the [0,0] columns. The G2D test (Nielsen *et al.*, 2005, 2009) consists in likelihood-ratio between a multinomial model including the likelihood of the observed frequency of the ***j***th genomic region given its empirical frequency under the demographic model (local probability) and a null multinomial model including the likelihood of the ***j***th genomic region given the genome-wide pattern of frequencies under the demographic model (global probability). This get written as

$$log(p(X^{(j)} \mid \widehat{P}^{\ (j)} \ / \ p(X^{(j)} \mid \widehat{P})$$

and since we are calculating G for a pair of populations, it can also be written as

$$G \ = \ 2(log(X^{(j)} \mid \widehat{P}^{\ (j)}\ ) \ - \ log(X^{(j)} \mid \widehat{P}) \ ,$$

with $X^{(j)}$ representing the data for the **j**th genomic region, and

$$\widehat{P}^{\ (j)}$$

the Maximum-Likelihood estimate of

$$\widehat{P}$$

considering only the data from the ***j***th genomic region. The G2D test measures the fit between the frequency of a genomic region and the genome-wide pattern of frequencies . The empirical background pattern of variation in the average 2D-SFSs provides the null model for the G2D test while the individual target region 2D-SFSs provide the alternative model. We proceeded with the same approach for the target regions and the windows and thus identified the largest G2D values as those exceeding the 95% quantile. We used the test on both levels to target (1) the genomic regions that differentiate at least one pair of populations, (2) the genomic regions

that differentiate all pair of populations, (3) the genomic regions which differentiate a population from all other

populations (*ie.* which pops up as outlier only when a population is paired with the target population) (WithAllAndOnlyPopXOutlierPopPairs), (4) genomic regions which are outlier when a pairing involves a target population (WithAllPopXOutlierPopPairs), and (5) genomic regions characterising a given pairing of populations (*ie.* which differentiate exclusively a given population from another) (WithAllAndOnlyPopXPopYOutlierPopPairs), and (6) genomic regions differentiating N3 and N4 from S1 and S5. The R scripts used to compute the G2D is the n°18.

Detailed methods for Bamova

Bamova tests whether a locus is over-differentiated (subject to divergent selection) with respect to the expected genomic (neutral) degree of differentiation. To this end, the software uses the Φ-statistics, a measure of partition of the molecular variation among populations which includes the genetic distance among haplotype sequences. The estimates of Φ-statistics were calculated for the haplotype frequencies at different hierarchy levels - both among groups (Φ_{CT}, clusters to total) and within groups (Φ_{SC}, population to cluster) - in order to capture the impact of slope, elevation and refuge on the sampling scheme. For each locus, Bamova draws estimates of Φ-statistics both from a common, genome-wide prior distribution, and the estimates of haplotypes frequencies and the genetic distances among haplotypes. Outlier identification of unusually high Φ-statistics values was based on the computation of a locus-specific Bayes Factor (BF), a point estimate of the parameter under the genomic model and the locus-specific model (its posterior probability) (Makowski *et al.*, 2019). Loci with a BF > 30 were considered outliers under "decisive evidence" (Jeffreys, 1998). Bamova was run under the known haplotype likelihood model. All the Bamova analyses were run with 1000000 MCMC iterations with a thinning interval of 100. The output included both genome-wide parameters and Φ-statistics.

Assessment of MCMC chains convergence for BayeScan and Bamova

We assessed the convergence of each Markov chain by assessing the evolution of the autocorrelation among iterations across each chain using Moran's I. We computed the index following the formula $N / W * (\Sigma_i\Sigma_j w_{ij} (x_i - \bar{\ }) * (x_j - \bar{\ }) / \Sigma i ((x_i - \bar{\ })^2),$ where N is the number of iterations in each chunk, W the sum of weights and w_{ij} the inverse matrix of the absolute value between two iterations, $iteration_i$ and $iteration_j$.

Using this method we discarded as burn-in 9000 samples for each pairwise dataset before analysing the remaining posteriors.

References

Gompert Z, Buerkle CA (2011). A hierarchical Bayesian model for next-generation population genomics. *Genetics* **187**: 903–17.

Jeffreys H (1998). *The theory of probability*. OuP Oxford.

Makowski D, Ben-Shachar MS, Chen SHA, Lüdecke D (2019). Indices of Effect Existence and Significance in the Bayesian Framework. *Front Psychol* **10**: 1–14.

Nielsen R, Hubisz MJ, Hellmann I, Torgerson D, Andrés AM, Albrechtsen A, *et al.* (2009). Darwinian and demographic forces affecting human protein coding genes. *Genome Res* **19**: 838–849.

Nielsen R, Williamson S, Kim Y, Hubisz MJ, Clark AG, Bustamante C (2005). Genomic scans for selective sweeps using SNP data. *Genome Res* **15**: 1566–1575.

Supplementary Methods 4

Adding custom genome to configuration file

In using SnpEff (Cingolani *et al.*, 2012), we edited the *SnpEff.config* file by adding the following line.

Bhaga.genome : fagus_sylvatica

The left part refers to the name of the fasta file containing the genomic sequence with the suffix *.genome* in place of *.fasta*/*.fa*. The right part describes the species the genome refers to.

Building genomic database with SnpEff

SnpEff requires a precise folder hierarchy to work: within the software folder, we created a “data” folder, containing both a “genomes” (with the reference genome, *Bhaga.fa*) and a “Bhaga” folder containing the gff3 file (*genes.gff*) and a fasta file of each CDS (*cds.fa*).

The latter twos were provided by by the repository of Mishra et al. (2022): the gff3 was originally named *Bhaga_genes.gff3*; the fasta file was instead named *Bhaga_genes.fna* and contained all the sequence of putative CDS in *F. sylvatica* genome.

We launched the following command line from the SnpEff folder to build the database:

```
java -jar snpEff.jar build -gff3 -v Bhaga -noCheckProtein
```

Running SnpEff to annotate a vcf file

The following commands were used to annotate the genomic and outlier vcf after having built the reference database:

```
java -Xmx4g -jar snpEff.jar -c snpEff.config -v Bhaga fagus96golden_genomic.vcf > fagus96golden_genomicAnnotated.vcf
```

```
java -Xmx4g -jar snpEff.jar -c snpEff.config -v Bhaga OutliersSNP_20122022.vcf > OutliersSNP_20122022_Annotated.vcf
```

References

Cingolani P, Platts A, Wang LL, Coon M, Nguyen T, Wang L, *et al.* (2012). A program for annotating and predicting the effects of single nucleotide polymorphisms, SnpEff: SNPs in the genome of Drosophila melanogaster strain w1118; iso-2; iso-3. *Fly (Austin)* **6**: 80–92.

Mishra B, Ulaszewski B, Meger J, Aury J-M, Bodénès C, Lesur-Kupin I, *et al.* (2022). A Chromosome-Level Genome Assembly of the European Beech (Fagus sylvatica) Reveals Anomalies for Organelle DNA Integration, Repeat Content and Distribution of SNPs. *Front Genet* **12**.

Supplementary Results 1

Genomic distribution of target regions

When tested for the conformity to a Poisson process; the genomic distribution of target regions provided a ratio of sample variance to sample mean of 10.81 (χ^2 test, 1 D.F., P-value = 0.001). Since a Poisson distribution mean and variance are equal, our findig exceeds the ratio of 1.0 and suggests overdispersion (i.e., sequenced genomic regions are more clustered than expected). This was expected, since the sequencing capture protocol targets specifically exomic regions, which are not uniformly distributed along the chromosomes.

Outlier search without controlling for the demographic background

Standard test selection based on population differentiation

Single-locus approach for outlier detection based on theoretical model of population differentiation

A locus is considered as an outlier whenever Bamova 1.02 (Gompert & Buerkle, 2011; Scotti et al., 2023) is a genome-based method for Φ-statistics outliers that compare the single-locus posterior distribution of the statistic to the genome-level posterior distribution. The analyses were carried over the six pairing of the four populations within an island framework. This "symmetrical" sampling scheme will allow us to reason on the outcomes by relying on the biogeographic and ecological differences within each pair. Details on the number of individuals and loci of each population pair are reported in Supplementary Table 2.

The method relies on the identification of outliers using Bayes Factors as a statistic to assess whether a given locus conforms to the null hypothesis of undergoing only the background observed genome-level divergence (Gompert & Buerkle, 2011; Scotti et al., 2023). The neutral expectations (generally speaking based on an island model divergence process) are therefore drawn from the observed data.

The pipelines used to obtain the respective input files and the detailed methods for outlier search are reported in Supplementary Methods 3.

"Crude" outlier search tests

Overall, 778 SNPs passed the significance tests of Bamova and were deemed as outliers: 535 pairwise tests and 243 tests of hierarchical schemes. When pooling hierarchical and pairwise

population analyses, the number of individual outlier loci was 562: 387 were retrieved under a single grouping and 175 by more than one, respectively. The 562 outlier SNPs were retrieved in 461 target regions. Among these regions, 389 harboured a single outlier SNP and 72 more than one.

The Bamova analyses retrieved **65** SNPs whose frequencies diverged with strong evidence from the genomic background between N3 and N4 populations (Φ_{ST} Northern slope), and **120** between S1 and S5 (Φ_{ST} Southern slope). When comparing population from the putative refugial area, the SNPs retrieved with a frequency strongly diverging from the background were **88** between N3 and S5, and **109** SNPs between N4 and S5 (Φ_{ST}). When the comparison was performed for population pairs belonging to different slopes and growing at different altitudes, Bamova retrieved **79** (N3-S1) and **74** (N4-S1) SNPs. When the full dataset is analysed within a hierarchical structure, we found **18** genomic outliers the number of genomic outliers diverging between the two slopes (Φ_{CT} slope replicates, "North vs. South"), another and **225** were divergent at different altitudes within the same slope (Φ_{SC} replicates "altitude").

References

Gompert Z, Buerkle CA (2011). A hierarchical Bayesian model for next-generation population genomics. *Genetics* **187**: 903–17.

Scotti I, Lalagüe H, Oddou□Muratorio S, Scotti□Saintagne C, Ruiz Daniels R, Grivet D, *et al.* (2023). Common microgeographical selection patterns revealed in four European conifers. *Mol Ecol* **32**: 393–411.

Supplementary Table 1

List of probe sequences used in the sequence capture experiment.

Please refer to stable repository at

https://entrepot.recherche.data.gouv.fr/dataset.xhtml?persistentId=doi:10.57745/BE4HIG

Supplementary Table 2

Number of SNPs for each stand pair after all the filtering steps and the subsetting.

**Pop: loci (inds)**	**N3 (20)**	**N4 (25)**	**S1 (25)**	**S5 (26)**
**N3 (20)**	-	31,159 (45)	30,966 (45)	31,121 (46)
**N4 (25)**		-	31,777 (50)	32,033 (51)
**S1 (25)**			-	31,729 (51)
**S5 (26)**				-

Supplementary Table 3

Starting range of the parameters for the FastSimCoal parameter estimation. Note that the signs of the GROWTHRATE parameter here reported must be flipped when writing a FastSimCoal inpout because the software package works within a coalescent framework, hence *backward* in time.

**Name**	**Description**	**min**	**max**	**distribution**
POPSIZE-N3	Current size of population N3	100	3000	log-uniform
POPSIZE-N4	Current size of population N4	100	3000	log-uniform
POPSIZE-S1	Current size of population S1	100	3000	log-uniform
POPSIZE-S5	Current size of population S5	50	3000	log-uniform
MIG	Symmetrical migration rate for all population pairs (independent draws for each population pair)	0.001	0.01	log-uniform
TIME1	Time of N3-N4 merger	1	TIMEA	log-uniform
TIME2	Time of S1-(N3-N4) merger	1	TIMEA	log-uniform
TIME3	TIme of final population merger (S5-(S1-(N3-N4)))	1	TIMEA	log-uniform
GROWTHRATE0	Growth rate until TIMEA	0.05	0.01	uniform
TIMEA	End of population contraction	2	20	log-uniform
TIMEB	End of population stability at minimum size	TIMEA	30	uniform
GROWTHRATE1	Growth rate until TIMEC	-10^{-9}	-0.001	log-uniform
TIMEC	End of population expansion	TIMEB	100	uniform
TIMED	Start of population contraction	TIMEC	300	uniform
GROWTHRATE2	Growth rate until TIMEE	10^{-9}	10^{-5}	log-uniform
TIMEE	End of population contraction	TIMED	1000	log-uniform

Supplementary Table 4

Comparison of the Observed and Simulated Divergence statistics. **Mean Obs** = Mean observed statistic; **Mean Sim** = Mean simulated statistic; **P-value** = P-value of the Wilcoxon test; **Wilcoxon test statistic**; **Bias** = type of bias of the test.

Diversity statistic	Stand pair	Mean Obs	Mean Sim	P-value	Wilcoxon Test statistic	Bias
ΔAllelic frequencies	N3-N4	0.044	0.048	2.2e-16	6962873554	Conservative
ΔAllelic frequencies	N3-S1	0.047	0.048	2.2e-16	7138465082	Conservative
ΔAllelic frequencies	N3-S5	0.046	0.048	2.2e-16	7128291932	Conservative
ΔAllelic frequencies	N4-S1	0.043	0.045	2.2e-16	7009882708	Conservative
ΔAllelic frequencies	N4-S5	0.045	0.045	0.7102	7344332686	Unbiased
ΔAllelic frequencies	S1-S5	0.045	0.045	0.1468	7301349790	Unbiased
G2D	N3-N4	14.02	17.39	0.7979	259939	Unbiased
G2D	N3-S1	14.25	17.38	0.9659	244064	Unbiased
G2D	N3-S5	14.32	17.53	0.9255	256201	Unbiased
G2D	N4-S1	14.73	18.11	0.9719	249405	Unbiased
G2D	N4-S5	15.04	18.24	0.9632	256923	Unbiased
G2D	S1-S5	15.07	18.24	0.9766	248905	Unbiased

Bamova $Φ_{ST}$	N3-N4	-1.11e-02	-1.11e-02	2.2e-16	629742486	Conservative
Bamova $Φ_{ST}$	N3-S1	-1.11e-02	-1.10e-02	2.2e-16	691267424	Conservative
Bamova $Φ_{ST}$	N3-S5	-1.08e-02	-1.08e-02	2.2e-16	794317267	Conservative
Bamova $Φ_{ST}$	N4-S1	-9.97e-03	-9.95e-03	2.2e-16	781706127	Conservative
Bamova $Φ_{ST}$	N4-S5	-9.75e-03	-9.74e-03	2.2e-16	809383688	Conservative
Bamova $Φ_{ST}$	S1-S5	-9.75e-03	-9.74e-03	2.2e-16	808016225	Conservative

Supplementary Table 5: Numbers of crude outliers

Bamova: number of non-demography corrected outliers. Müller *et al* (2013)*;* Lalagüe / Csilléry *et al.,* (2014)*;* Lesur *et al.,* (2015)*;* Postolache et al. (2021); Meger et al. (2021): numbers of Bamova outliers that are also outliers in the respective studies (see main document); numbers in parentheses show the genomic regions in common between the Bamova outliers and the regions targeted in each study. So for example, in N4-S5, two sequences were in common between the 109 Bamova outliers and the sequences studied in Lalagüe / Csilléry; one of these is an outlier in both analyses.

Stand pair	Bamova	Müller	Lesur	Postolache	Lalagüe / Csilléry	Meger
N3-N4	65	2 (59)	0 (64)	0 (0)	0 (0)	3 (3)
N3-S1	79	4 (77)	1 (79)	0 (0)	0 (0)	0 (5)
N3-S5	88	0 (73)	0 (87)	0 (0)	0 (0)	0 (0)
N4-S1	74	3 (70)	0 (73)	1 (1)	0 (0)	0 (0)
N4-S5	109	1 (93)	0 (108)	0 (0)	1 (2)	0 (1)
S1-S5	120	1 (114)	0 (119)	0 (0)	0 (0)	1 (1)

References

Csilléry K, Lalagüe H, Vendramin GG, González-Martínez SC, Fady B, Oddou-Muratorio S (2014). Detecting short spatial scale local adaptation and epistatic selection in climate-related candidate genes in European beech (Fagus sylvatica) populations. *Mol Ecol* **23**: 4696–4708.

Lesur I, Le Provost G, Bento P, Da Silva C, Leplé JC, Murat F, *et al.* (2015). The oak gene expression atlas: Insights into Fagaceae genome evolution and the discovery of genes regulated during bud dormancy release. *BMC Genomics* **16**.

Meger J, Ulaszewski B, Burczyk J (2021) Genomic signatures of natural selection at phenology-related genes in a widely distributed tree species Fagus sylvatica L. *BMC Genomics* **22**: 1-20.

Müller M (2013). A candidate gene-based association study to investigate potentially adaptive genetic variation in European beech (*Fagus sylvatica* L.). Göttingen University.

Postolache D, Oddou-Muratorio S, Vajana E, Bagnoli F, Guichoux E, Hampe A, *et al.* (2021). Genetic signatures of divergent selection in European beech (Fagus sylvatica L.) are associated with the variation in temperature and precipitation across its distribution range. *Mol Ecol*: mec.16115.

References for the scripts

Excoffier L, Foll M (2011). fastsimcoal: A continuous-time coalescent simulator of genomic diversity under arbitrarily complex evolutionary scenarios. *Bioinformatics* **27**: 1332–1334.

Goudet J (2005). HIERFSTAT, a package for R to compute and test hierarchical F-statistics. *Mol Ecol Notes* **5**: 184–186.Jombart T (2008). Adegenet: A R package for the multivariate analysis of genetic markers. *Bioinformatics* **24**: 1403–1405.

Jost L (2007). Partitioning diversity into independent alpha and beta components. *Ecology* **88**: 2427–2439.

Jost L (2008). Gst and its relatives do not measure differentiation. *Mol Ecol* **17**: 4015–4026.

Nielsen R, Hubisz MJ, Hellmann I, Torgerson D, Andrés AM, Albrechtsen A, *et al.* (2009). Darwinian and demographic forces affecting human protein coding genes. *Genome Res* **19**: 838–849.

Nielsen R, Williamson S, Kim Y, Hubisz MJ, Clark AG, Bustamante C (2005). Genomic scans for selective sweeps using SNP data. *Genome Res* **15**: 1566–1575.
